# Dual Fatty Acid Synthase and HER2 Signaling Blockade Shows Marked Antitumor Activity against Breast Cancer Models Resistant to Anti-HER2 Drugs

**DOI:** 10.1371/journal.pone.0131241

**Published:** 2015-06-24

**Authors:** Adriana Blancafort, Ariadna Giró-Perafita, Glòria Oliveras, Sònia Palomeras, Carlos Turrado, Òscar Campuzano, Dolors Carrión-Salip, Anna Massaguer, Ramon Brugada, Marta Palafox, Jorge Gómez-Miragaya, Eva González-Suárez, Teresa Puig

**Affiliations:** 1 New Therapeutic Targets Lab (TargetsLab), Department of Medical Sciences, Faculty of Medicine, University of Girona, Girona, Spain; 2 Catalan Institute of Oncology, Hospital Dr. Josep Trueta, Girona, Spain; 3 Química Orgánica I, Facultad de Ciencias Químicas, Universidad Complutense, Madrid, Spain; 4 Cardiovascular Genetics Center, University of Girona-IDIBGi, Girona, Spain; 5 Biochemistry and Molecular Biology Unit, Department of Biology, University of Girona, Girona, Spain; 6 Cancer Epigenetics and Biology Program (PEBC), Bellvitge Institute for Biomedical Research (IDIBELL), Hospitalet de Llobregat-Barcelona, Spain; University of Algarve, PORTUGAL

## Abstract

Blocking the enzyme Fatty Acid Synthase (FASN) leads to apoptosis of HER2-positive breast carcinoma cells. The hypothesis is that blocking FASN, in combination with anti-HER2 signaling agents, would be an effective antitumor strategy in preclinical HER2+ breast cancer models of trastuzumab and lapatinib resistance. We developed and molecularly characterized *in vitro* HER2+ models of resistance to trastuzumab (SK**TR**), lapatinib (SK**LR**) and both (SK**LTR**). The cellular interactions of combining anti-FASN polyphenolic compounds (EGCG and the synthetic G28UCM) with anti-HER2 signaling drugs (trastuzumab plus pertuzumab and temsirolimus) were analyzed. Tumor growth inhibition after treatment with EGCG, pertuzumab, temsirolimus or the combination was evaluated in two *in vivo* orthoxenopatients: one derived from a HER2+ patient and another from a patient who relapsed on trastuzumab and lapatinib-based therapy. SK**TR**, SK**LR** and SK**LTR** showed hyperactivation of EGFR and p-ERK1/2 and PI3KCA mutations. Dual-resistant cells (SK**LTR**) also showed hyperactivation of HER4 and recovered levels of p-AKT compared with mono-resistant cells. mTOR, p-mTOR and FASN expression remained stable in SK**TR**, SK**LR** and SK**LTR**. *In vitro*, anti-FASN compounds plus pertuzumab showed synergistic interactions in lapatinib- and dual- resistant cells and improved the results of pertuzumab plus trastuzumab co-treatment. FASN inhibitors combined with temsirolimus displayed the strongest synergistic interactions in resistant cells. *In vivo*, both orthoxenopatients showed strong response to the antitumor activity of the combination of EGCG with pertuzumab or temsirolimus, without signs of toxicity. We showed that the simultaneous blockade of FASN and HER2 pathways is effective in cells and in breast cancer models refractory to anti-HER2 therapies.

## Introduction

The human epidermal growth factor receptor 2 (HER2) is amplified or overexpressed in ∼ 20% of human breast carcinomas and is associated with a more aggressive phenotype and worse prognosis [1].

HER receptors family is composed of four closely related tyrosine kinase (TK) receptors: HER1 (EGFR), HER2, HER3, and HER4. Dimerization of HER receptors, induced by ligand binding or receptor overexpression in the case of HER2, leads to the recruitment of several adaptor proteins that mediate the activation of downstream signaling pathways [2, 3]. Among them, the phosphoinositide 3-kinase (PI3K)/protein kinase B (PKB/AKT)/mammalian target of rapamycin (mTOR) protein and the mitogen activated protein kinases (MAPK or ERK1/2) pathways promote cell proliferation, transformation, and survival [4, 5].

HER2-overexpressing tumors are sensitive to monoclonal antibodies (mAb) and small-molecule TK inhibitors (TKI) that interfere with HER2 function and signaling [6–8]. Trastuzumab, a humanized mAb directed against the extracellular domain of the receptor, was the first approved therapy for the treatment of HER2-positive (HER2+) breast cancer. Despite the considerable clinical benefit provided, a large fraction of HER2+ tumors display primary or acquired resistance to trastuzumab [9]. Lapatinib, a small-molecule TKI targeting the intracellular tyrosine kinase domain of EGFR and HER2, was found to improve time to progression in HER2 breast cancer patients who had progressed to tratuzumab [7]. Lapatinib is administered alone or in combination with trastuzumab to abolish the activation of HER2-downstream pathway. But unfortunately, some tumors develop lapatinib resistance and also resistance against the combination of both drugs [10]. The molecular mechanisms leading to trastuzumab and lapatinib resistance has been extensively studied [11]. These include for example *in vivo* conversion of HER2+ to HER2- carcinoma after neoadjuvant trastuzumab [12], predominance of the constitutively active HER2 form (p95^HER2^) [8], overexpression or hyperactivation of other HER family receptors or its ligands [13], amplification of the PI3K/AKT/mTOR pathway by loss of phosphatase and tensin homolog (PTEN) [14], gain-of-function mutation in PI3KCA (encoding the PI3K catalytic isoform p110α) [15] and AKT mutations or amplifications [16].

Fatty acid synthase (FASN) is a homodimeric multienzymatic protein that catalyzes de novo synthesis of long-chain fatty acids [17]. Blocking FASN activity causes *in vitro* and *in vivo* anticancer activity in several overexpressing FASN human carcinomas [18, 19]. The proposed oncogenic properties of FASN seem to be the result of an increased activation of HER2 and its dowstream related PI3K/AKT/mTOR and MAPK signaling pathways [18–20]. FASN can also inhibit the intrinsic pathway of apoptosis [21], may also contribute to modulation of the membrane lipid rafts that anchor HER2 [22] and has been recently proposed as a direct target of p53 family members, including p63 and p73 [23]. In the past, FASN inhibitors with antitumour activity have been limited by either cross-activation of β-oxidation, which produces *in vivo* anorexia and body weight loss [24, 25], or low potency [26, 27]. We have developed new polyphenolic anti-FASN compounds that exhibit *in vitro* and *in vivo* anticancer activity improving the antitumor efficacy and the toxic effects of classical FASN inhibitors, in HER2+ breast cancer cells and mouse models [19, 28, 29]. Among of them, G28UCM has shown a strong antitumor effect, alone or in combination with anti-HER drugs, in HER2+ breast cancer cells and on breast cancer cells resistant to trastuzumab [29].

In this study, we have investigated the anticancer activity of the classical FASN inhibitor epigallocathequin-3-gallate (EGCG) and G28UCM, as single agents or in combination with pertuzumab and temsirolimus, in our developed trastuzumab (SK**TR**), lapatinib (SK**LR**) and trastuzumab *plus* lapatinib (SK**TLR**) resistant HER2+ breast cancer models. In addition, we analyzed the antitumor activity of EGCG, alone or in combination, in two *in vivo* xenografts: one HER2+ patient and another from a HER2+ patient who fail to respond to trastuzumab and lapatinib therapies.

## Materials and Methods

### Cell culture and development of long-term resistant breast cancer cells

SKBr3 (SK) breast carcinoma cells were obtained from Eucellbank (University of Barcelona) [30]. SKBr3 cells were routinely grown in McCoy’s (Gibco) supplemented with 10% FBS (HyClone Laboratories), 1% L-glutamine, 1% sodium pyruvate, 100 U/mL penicillin, and 100 μg/mL streptomycin (Gibco). Trastuzumab-resistant SK cells (SK**TR**) were developed by exposing SK cells continuously to trastuzumab (Herceptin, Hoffmann-La Roche Pharma), starting with 1μM concentration for three months of exposure and increasing the concentration up to 2 μM for a 12 months period, as we previously described [29]. Thus, cells resistant to trastuzumab were maintained in 2 μM trastuzumab, a concentration at which SK parental cells were not viable. To develop lapatinib-resistant cells (SK**LR**), SK cells were treated for one month with an initial dose of 1.5 μM of lapatinib (GW572016; Tykerb, GlaxoSmithKline) and after one month the dose of lapatinib was increased up to 3 μM for 12 months as we described [29], a concentration at which SK parental cells were not viable. To develop lapatinib plus trastuzumab resistant cells (SK**LTR**), SK**LR** were co-cultured with lapatinib 3 μM and trastuzumab 1μM and after one month in culture the dose of trastuzumab was increased up to 2 μM. Cells were co-cultured with lapatinib and trastuzumab for 12 months. SK**LTR** cells were mantained with 3 μM of lapatinib and 2 μM of trastuzumab. Trastuzumab, lapatinib and trastuzumab plus lapatinib resistance was confirmed by dose-response studies using the standard colorimetric MTT assay as we describe in S1 File. Cell line authentication was performed with STR analysis in an external laboratory (Genetica DNA Laboratories) (S2 File). Parental and resistant cells shared 100% STR profile with SKBr3 cell line.

### HER2-Fluorescent *in situ* hybridization (FISH)

HER2 FISH pharmDX Kit (Dako) was used to quantify HER2 gene copy number in parental and resistant cells as previously described [29]. The ratio of average HER2 to average CEN17 copy number was calculated for twenty nuclei. Gene amplification was defined when the FISH ratio HER2 signal / CEN17 signal was > 2.

### Western blot analysis of tumor and cell lysates

Parental (SK) and resistant (SKTR, SKLR and SKLTR) cells were serum-deprived for 24 hours in 0.5% FBS-medium, then were lysed with ice-cold in lysis buffer (Cell Signaling Technology, Inc.) containing 1 mM EDTA, 150 mM NaCl, 100 μg/mL PMSF, 50 mM Tris-HCl (pH 7.5), protease and phosphatase inhibitor cocktails (Sigma). Equal amounts of protein were heated in LDS Sample Buffer and Sample Reducing Agent (Invitrogen) for 10 min at 70°C, separated on SDS-polyacrylamide gel (SDS-PAGE), and transferred to nitrocellulose membranes. Blots were incubated overnight at 4°C with the following primary antibodies: rabbit polyclonal antibodies against FASN (Assay Designs; 905-069; dilution 1:1500), HER2/ErbB2/Neu (C-18) (Santa Cruz Biotechnology Inc.; SC-284; dilution 1:1000), EGFR (Cell Signaling Technology Inc.; #2232; dilution 1:200), phospho-EGFR^Tyr1068^ (Cell Signaling Technology Inc.; #2234; dilution 1:200), AKT (Cell Signaling Technology Inc.; #9272; dilution 1:500), p44/42 MAPK (Erk 1/2) (Cell Signaling Technology Inc.; #9102; dilution 1:500), and phospho-mTOR^Ser2448^ (Cell Signaling Technology Inc.; #2971; dilution 1:500); rabbit monoclonal antibodies against HER3/ErbB3 (Cell Signaling Technology Inc.; #4754; dilution 1:200), phospho-HER3/ErbB3^Tyr1289^ (Cell Signaling Technology Inc.; #4791; dilution 1:200), HER4/ErbB4 (Cell Signaling Technology Inc.; #4795; dilution 1:200), phospho-HER4/ErbB4^Tyr1284^ (Cell Signaling Technology Inc.; #4757; dilution 1:200), phospho-AKT^Ser473^ (Cell Signaling Technology Inc.; #4058; dilution 1:200) and mTOR (Cell Signaling Technology Inc.; #2983; dilution 1:500), and mouse monoclonal antibodies against phospho-p44/42 MAPK (Erk 1/2)^Thr202/Tyr204^ (Cell Signaling Technology Inc.; #9106; dilution 1:500) and phospho-c-erbB-2 (HER-2/neu)^Tyr1248^ (Thermo Scientific Inc.; MS-1072-P1; dilution 1:200). Antibodies were diluted in blocking buffer (2.5% powdered-skim milk in phosphate buffered saline solution with 0.05% Tween 20, PBS-T (10 mM Tris-HCL pH 8,0 and 150 mM NaCl). Then, blots were incubated with mouse and rabbit peroxidase-conjugated secondary antibody and revealed using a commercial kit (Super Signal West Pico or Super Signal West Femto chemiluminescent substrate (Thermo Scientific Inc.) or Immobilon Western HRP Substrate (Millipore). Blots were re-proved with a mouse monoclonal antibody against β-actin (Santa Cruz Biotechnology Inc.) as control of protein loading and transfer. Western blot analyses were repeated at least three times and representative results are shown (S3 File).

### Genetic analysis of PI3K mutations

DNA was extracted from SK, SK**TR**, SK**LR** and SK**LTR** cells following commercial protocols (QIAamp DNA blood Mini kit, Qiagen). Subsequently, polymerase chain reaction (PCR) was used to amplify the *PI3K* gene (NM_006218) (NCBI-National Center for Biotechnology Information). PCR products were purified using ExoSAP-IT (Isogen Life Science), and the analysis of the exonic and intron-exon regions was performed forward/reverse by direct sequencing (Genetic Analyzer 3130XL, Applied Biosystems).

### Quantitative real-time PCR analysis of HER ligands

Parental and resistant cells were washed with PBS and trypsinized. Total-RNA from each sample was isolated using RNeasy mini kit (Qiagen). RNA was reverse-transcribed into complementary DNA (cDNA) using High Capacity cDNA Archive Kit (Applied Biosystems). HER ligands expression (EGF, TGF-α, AR, BTC, EREG, NRG-1 and HB-EGF) was quantified by real-time PCR using a pre-designed, gene-specific TaqMan probe and primer sets (TaqMan Gene Expression assays, Applied Biosystems). Quantitative PCR was performed using TaqMan One-Step Universal Master Mix (Applied Biosystems) and 7300 Real-Time PCR system (Applied Biosystems). All samples were tested in triplicate. Relative quantification of the mRNA level (μg/ml) of HER ligands was carried out. Then, mRNA level was normalized to the housekeeping gene TATA box binding (TBP) protein.

### Cell invasion and adhesion assays

Parental and resistant cells were overnight FBS-starved (0.5% FBS-medium) before carrying the CytoSelect 24-well cell invasion assay and the CytoSelect 48-well cell adhesion assay (Cell Biolabs), following the manufacturer’s instructions.

### Growth inhibition and dose-response studies

Parental and resistant cells were plated out at a density of 5 x 10^3^ cells/100 μL/well in 96-well microtitre plates. Following overnight cell adherence fresh medium along with the corresponding concentrations of HER2 inhibitors (trastuzumab and pertuzumab [2C4, Perjeta, Genentech]), FASN inhibitors (EGCG [Sigma] and 1,3-bis((3,4,5-thilhydroxybenzoil)oxy)naphthalene (G28UCM) synthesized as we previously described [19]) or mTOR inhibitor (temsirolimus; CCI-779, Torisel, Pfizer) was added to the cultures. Pertuzumab (5 μg/ml) was combined with trastuzumab (20 μM) or FASN inhibitors (60 μM of EGCG or 5 μM of G28UCM) for 5 days. For temsirolimus drug-combination experiments cells were treated for 2 days with a dose curve concentration of EGCG (5–300 μM) or G28UCM (0.1–15 μM) plus fixed concentrations of temsirolimus (0.05, 0.1, 0.5 and 1 μM). Same treatments were assessed in monotherapy. Following treatment, media was replaced by drug-free medium (100 μL/well) containing MTT (3,4,5-dimethylthiazol-2-yl-2,5-diphenyltetrazolium bromide, Sigma) solution, and incubation was prolonged for 3 h at 37°C. Formazan crystals formed by metabolically viable cells were dissolved in DMSO (100 μL/well) and absorbance was determined at 570 nm in a multi-well plate reader (Model Anthos Labtec 2010 1.7). Using control OD values (*C*) and test OD values (*T*), % of cell proliferation inhibition (% cpi) was calculated from the equation, 100 - [(T x 100) / C]. Data presented are from three separate wells per assay and the assay was performed at least three times. Combinatorial effects were evaluated using the ratio of % cpi produced by each drug alone *vs* % cpi produced by drug combination (% cpi drug / % cpi combination), average of both ratios was calculated to know the effect of combination compared with both compounds alone. Interactions of G28UCM and EGCG with temsirolimus were also evaluated by the isobologram method as we previously published [29]. Briefly, the concentration of one agent producing a 30% inhibitory effect is plotted on the horizontal axis, and the concentration of another agent producing the same degree of effect is plotted on the vertical axis; a straight line joining these two points represents zero interaction (addition) between two agents. The experimental isoeffect points were the concentrations (expressed relative to the IC_**30**_ concentrations) of the two agents that when combined kill 30% of the cells. When the experimental isoeffect points felt below that line, combination effect of the 2 drugs was considered to be supra-additive or synergistic, whereas antagonism occurs if the experimental isoeffect points lie above it. Within the designed assay range, a set of isoeffect points was generated because there were multiple FASN inhibitors and anti-target agent concentrations that achieved the same isoeffect. A quantitative index of these interactions was provided by the equation Ix = (A/a) + (B/b), where, for this study, a and b represent the respective concentrations of FASN inhibitors (EGCG or G28UCM) and anti-mTOR agent (temsirolimus) required to produce a fixed level of inhibition (IC_**30**_) when administered alone, and A and B represent the concentrations required for the same effect when the drugs were administered in combination, and Ix represents an index of drug interaction (interaction index). Ix values of <1 indicate synergism, a value of 1 represents additivism, and values of >1 indicate antagonism. For all estimations of Ix, we used only isobolos where intercept data for both axes were available.

### *In vivo* studies: human breast tumor PDX (patient-derived xenografts) experiments

Tumor chunks from HER2+ breast cancer patient and HER2+ patient who relapsed after trastuzumab and lapatinib-based treatment were orthotopically implanted into both inguinal cleared mammary fat pads of NOD/SCID (Harlan Laboratories, Inc.) or NSG (NOD/SCID;IL2Rγ−/−) mice (Charles River Laboratories). When tumors reached 30–60 mm^3^, animals were randomized into treatment-groups. Each group received a single intraperitoneal (i.p.) injection (maximum of 0.2 mL) of control (vehicle alone 3d/wk), 30 mg/kg EGCG 3d/wk, 30 mg/kg pertuzumab 1d/w, 10 mg/kg temsirolimus 1d/w, or combination of EGCG + pertuzumab and EGCG + temsirolimus. Tumor xenografts were measured with calipers and tumor volumes were determined using the formula: (π/6 x (v1 x v2 x v2)), where v1 represents the largest tumor diameter, and v2 the smallest one. Body weight was registered 2–3 d/wk. At the end of the experiment, animals were weighed and then euthanized using CO_2_ inhalation. Tumors and serum were stored at -80°C. Lung, heart, liver, kidneys and tumor were fixed with formalin (S1 File). Apoptosis in control and treated tumors was analyzed by fluorescent TUNEL assay. Briefly, DNA fragmentation by terminal deoxynucleotidyl transferase-mediated dUTP-biotin nickend labeling (TUNEL) was performed according to the manufacturer’s instructions (in situ cell death detection, Roche). Nuclei contrast was performed using fluorescent DAPI staining (4′-6′-Diamidino-2- phenylindole, Sigma). Pictures shown are representative of two samples per each treatment group.

### Ethics Statement

Experiments were conducted in accordance with guidelines on animal care and use established by Biomedical Research Institute of Bellvitge (IDIBELL) Institutional Animal Care and Scientific Committee (protocol EST-FOR-070.03). All mice were maintained in a specific pathogen-free AAALAC (Association for Assessment and Accreditation of Laboratory Animal Care International) International accredited facility in accordance with Spanish and European regulations with controlled light/dark cycle, temperature, and humidity. As approved by the above-mentioned committee, all surgery was performed under inhaled isoflurane anesthesia, burprenorphine was administered as analgesic to mice after surgery, all mice were euthanized with CO_2_ asphyxiation at the end of the experiment or when tumors reached 1 cm of diameter, and all efforts were made to minimize suffering.

### Statistical analysis

Data were analyzed by Student *t* test when comparing two groups or ANOVA using a Bonferrony test as post-test when comparing more than 2 groups. Statistical significant levels were p < 0.05 (denoted as *), p < 0.01 (denoted as **) and p < 0.001 (denoted as ***). *p-value* is shown in results when significance is reached (p < 0.05). All data are means ± standard error (SE). All observations were confirmed by at least three independent experiments.

## Results

### Characterization of trastuzumab (SKTR), lapatinib (SKLR) and lapatinib plus trastuzumab-resistant (SKLTR) breast cancer cells

**Fig 1:**
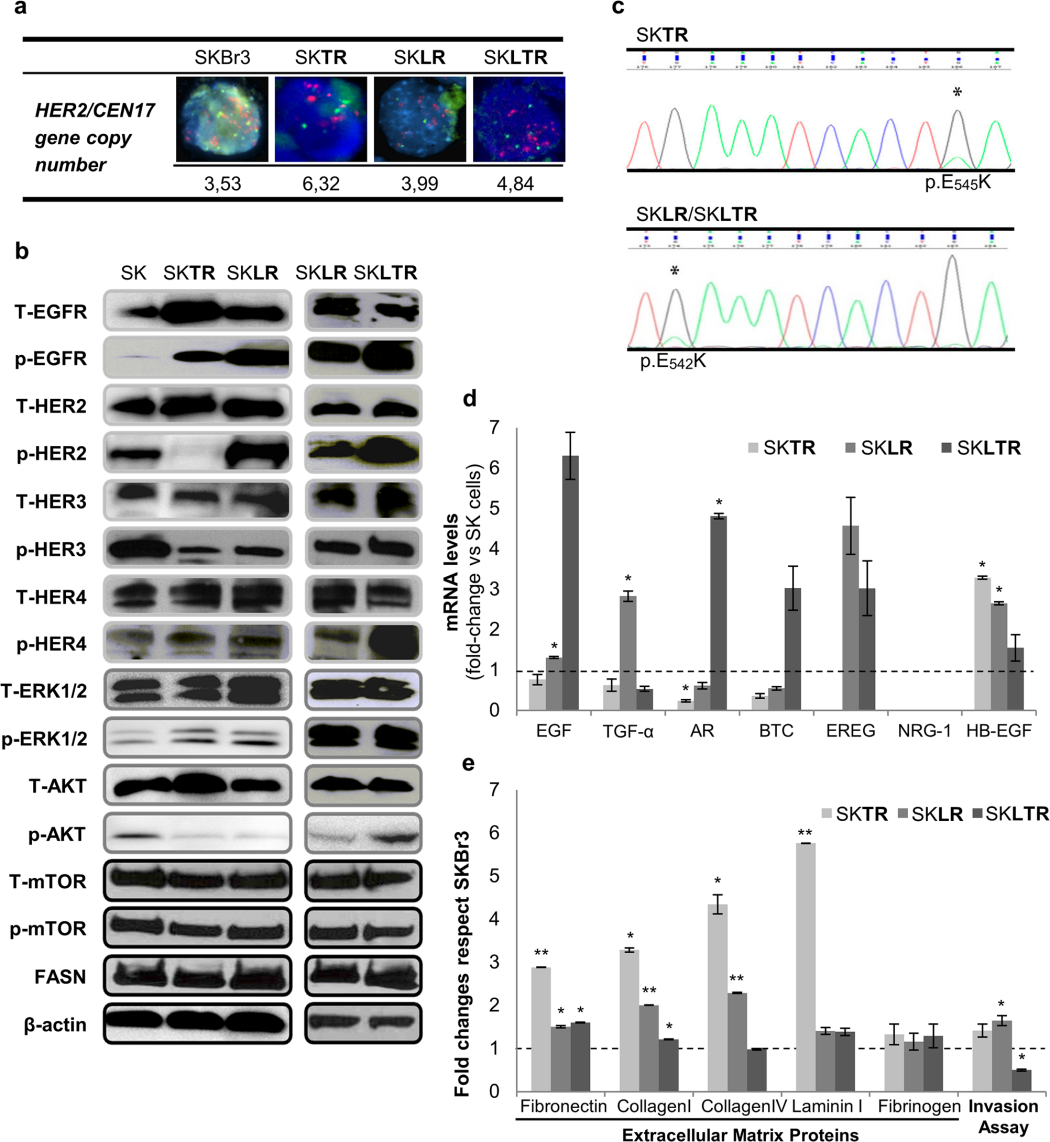
Characterization of parental (SK) and resistant (SKTR, SKLR and SKLTR) cells. **(a)** HER2 gene copy number is maintained in resistant cells. FISH, fluorescence in situ hybridization; HER2/CEN17 > 2 indicates HER2 gene amplification. **(b)** Resistant cells showed changes in the expression and activation of EGF family receptors but maintained downstream pathways activation (ERK1/2/AKT/mTOR) without affecting FASN protein expression levels. Protein expression and activation of EGFR family receptors pathways was analyzed by Western Blot. Gels shown are representative of those obtained from 3 independent experiments. **(c)** Mutational status of PIK3CA gene in resistant cells. Trastuzumab-resistant cells (SK**TR**) acquire the activating PIK3CA_E545K mutation and lapatinib- and lapatinib plus trastuzumab-resistant cells (SK**LR** and SK**LTR**) acquire the activating PIK3CA_E542K mutation. DNA sequencing of PI3K exon 9 of the resistant cells compared with the parental cells. **(d)** EGFR ligands are increased in resistant cells. Changes in the expression of each ligand by acquisition of resistance were assessed by real-time PCR and values were normalized against the corresponding mRNA expression of TBP constitutive gene. Then, ligands expression of trastuzumab-, lapatinib- and trastuzumab plus lapatinib-resistant cells (SK**TR**, SK**LR**, SK**LTR**) was compared to parental cells (SK). The bars indicate the mean fold change ± SE of two independent quantifications. Bars over the dotted line indicate an increase in the gene expression compared to the control cells, while bars under the dotted line represent impaired gene expression after the treatment. **(e)** Cellular adhesion and invasion capacity are increased in resistant cells. Fold-changes of resistant cells (SK**TR**, SK**LR** and SK**LTR**) respect to wild type SKBr3 cells (SK) in adhering to extracellular matrix proteins or in invasion capacity. Fold changes were assessed with adhesion or invasion kit assays. Experiments were performed at least twice. * (p ≤ 0.05) and ** (p ≤ 0.01) indicate levels of statistically significant difference compared with parental cells.

As preclinical models of acquired resistance to anti-HER2 drugs, we developed a panel of resistant HER2+ breast cancer cells (SK) with long-term (12 months) and high drug concentration exposure of trastuzumab (SK**TR**), lapatinib (SK**LR**) and lapatinib *plus* trastuzumab (SK**LTR**) (S1 Fig), following Nahta R *et al*. methodology [31]. To elucidate molecular mechanisms regarding acquired resistance in our developed resistant cells (SK**TR**, SK**LR** and SK**LTR**) we first examined HER2 gene amplification by fluorescence *in situ* hybridation. The ratio of the average HER2 gene copy number to the average CEP17 gene copy number in SK was 3.53 and in SK**TR**, SK**LR** and SK**LTR** was 6.32, 3.99 and 4.84, respectively (Fig 1A). These results showed that resistant cells possess HER2 amplification, similar as parental cells.

We next analyzed changes in HER family protein receptors and their downstream proteins related to PI3K/AKT/mTOR and MAPK/ERK1/2 pathways. As shown in Fig 1B, trastuzumab resistant cells (SK**TR**) had a significant increase in EGFR and phosphorylated EGFR proteins and to a lesser extent in p-ERK1/2 and AKT, with a noticeably decreased p-HER2 protein and reduced levels of HER3, p-HER3 and p-AKT compared to SK control cells. Cells resistant to lapatinib (SK**LR**) showed a great increase in p-EGFR and p-HER2 proteins and a slightly increase in p-ERK1/2, whereas levels of HER3, p-HER3 and p-AKT were decreased compared to SK control cells. Lapatinib *plus* trastuzumab-resistant cells (SK**LTR**) showed increased expression of phosphorylated forms of HER2, EGFR, HER4 and AKT compared to its control lapatinib-resistant cells (SK**LR**). Interestingly, no significant changes in mTOR and p-mTOR protein levels were observed in any resistant cells compared to SK cells. Regarding FASN, which transcription and translation is mediated by HER2 signaling pathway [29, 32], protein expression levels showed no changes in resistant cells.

Mutation of PI3K is another described mechanism of resistance to anti-HER2 treatments. We found low incidence of activating mutations in the p110α catalytic subunit of PI3K (*PIK3CA)* in all resistant cells. We detected the PIK3CA_E545K mutation in SK**TR** cells and the PIK3CA_E542K mutation in SK**LR** and its derivative SK**LTR** (Fig 1C).

Changes in crosstalk between receptors of HER family prompted us to investigate the mRNA expression profile of several HER activating ligands by real-time PCR in resistant and parental cells (Fig 1D). HB-EGF (heparin-binding EGF-like growth factor) mRNA expression was significantly up-regulated (more than 3-fold; *p-value*: *0*.*012*) in trastuzumab resistant cells (SK**TR)** compared to SK control cells. In contrast, AR (amphiregulin; *p-value*: *0*.*024*) was down-regulated, and TGF-α (transforming growth factor-α) and BTC (beta-cellulin) expression was slightly down-regulated in SK**TR** *versus* SK cells. Otherwise, SK**LR** cells up-regulated EGF (epidermal growth factor) (1.5 folds compared with SK; *p-value*: *0*.*045*), TGF-α (nearly 3 folds; *p-value*: *0*.*044*), EREG (epiregulin) (4.5 folds) and HB-EGF (more than 2.5 folds; *p-value*: *0*.*014*), but down-regulated AR and BTC expression in about 0.5 folds, comparing with SK. Double-resistant (SK**LTR**) cells showed a great up-regulation in almost all ligands (EGF in more than 6 folds, AR in almost 5 folds (*p-value*: *0*.*011)*, BTC and EREG in 3 folds and HB-EGF almost 2 folds), only TGF-α was slightly down-regulated in 0.5 folds, comparing with SK cells.

Since some studies reported that HER2 mediates tumor growth and metastasis [33, 34], and several molecular changes (that could alter tumor aggressiveness) occurred on HER2 and its downstream pathways, we conducted experiments to evaluate such hallmark by measuring cell invasion and adhesion to extracellular matrix (opening metastatic event) capacities of our developed resistant cells (Fig 1E). Moreover, several types of resistance such as some chemotherapeutic drugs and multidrug resistance (combining several natural chemotherapeutic drugs) have been associated with cancer invasion and metastasis [35, 36]. Hence, we wanted to know if mechanisms of resistance in our anti-HER2 resistant models also turned to a more aggressive phenotype of tumor cells. SKTR cells showed a huge significantly increased capacity to adhere to extracellular matrix proteins: fibronectin (2.9 fold-change; *p-value*: *0*.*002*), collagen I (3.3 fold-change; *p-value*: *0*.*013*), collagen IV (4.4 fold-change; *p-value*: *0*.*043*), laminin I (5.8 fold-change; *p-value*: *0*.*001*) and a slightly increase in fibrinogen adherence (1.3 fold-change) compared with SK parental cells. SK**LR** cells had a remarkable increased adherence to fibronectin (1.5 fold-change; *p-value*: *0*.*031*), collagen I (2 fold-change; *p-value*: *0*.*003*) and collagen IV (2.3 fold-change; *p-value*: *0*.*005*) and to a lesser extent to laminin I (1.4 fold-change) and fibrinogen (1.1 fold-change) compared with SK cells. SK**LTR** showed a significant increased capacity to adhere to fibronectin (1.6 fold-change; *p-value*: *0*.*011*) and collagen I (1.2 fold-change; *p-value*: *0*.*030*), a slight adherence to laminin I (1.4 fold-change) and fibrinogen (1.3 fold-change), whereas adhesion to collagen IV was almost unchanged, compared to SK. Regarding the invasion capacity, both SK**TR** and SK**LR** cells showed relevant increased capacity than SK cells [1.4 and 1.6 (*p-value*: *0*.*011*) folds more, respectively]. Conversely, SK**LTR** cells showed half invasion capacity (0.5 folds; *p-value*: *0*.*025*) than SK cells (Fig 1E).

### *In vitro* cell growth interactions between HER2 inhibitors, trastuzumab and pertuzumab, in SK, SKTR, SKLR and SKLTR

**Fig 2:**
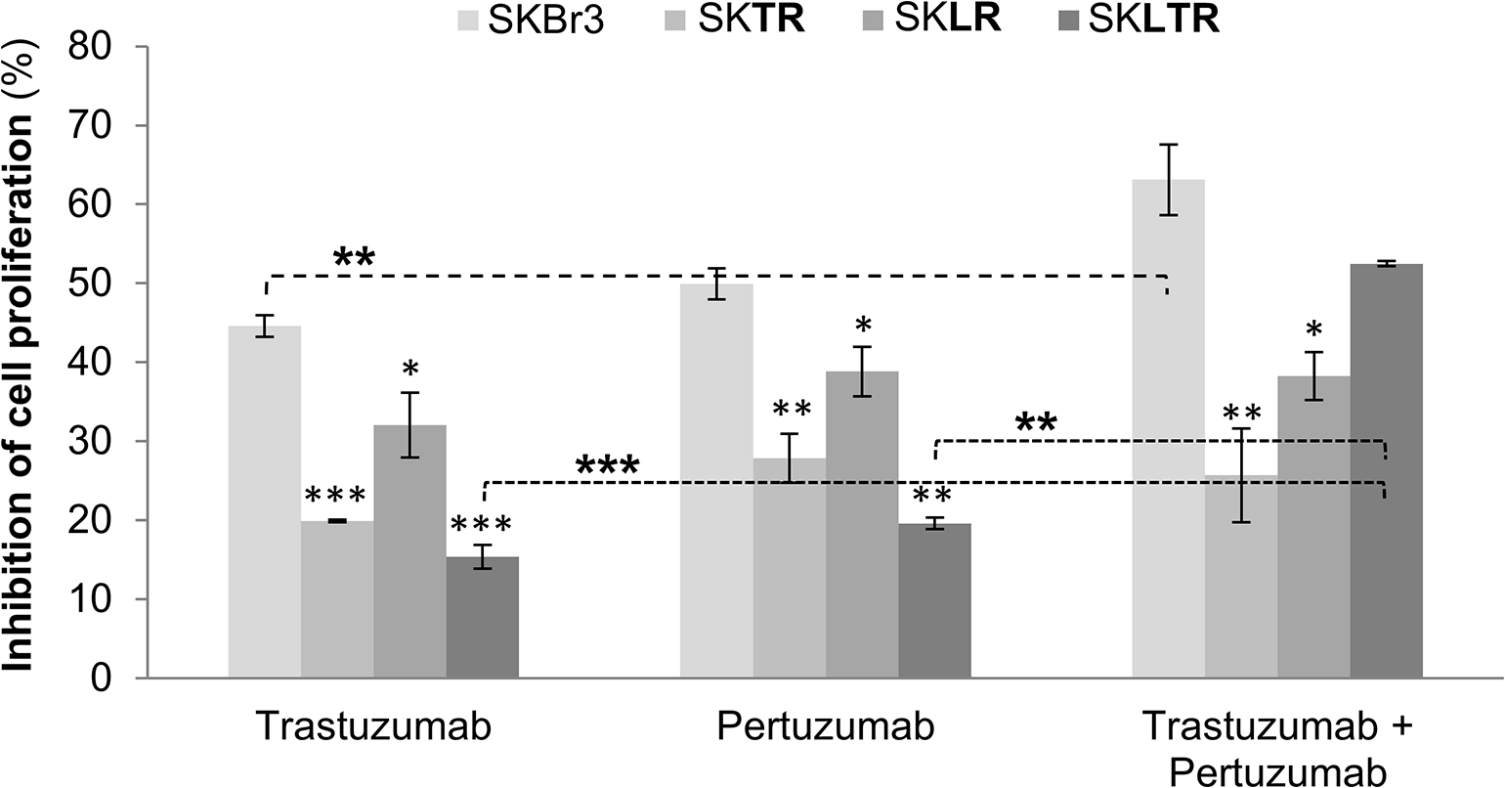
Pertuzumab plus trastuzumab combination improves effects in SK and SKLTR. Cells were treated with trastuzumab (20 μM), pertuzumab (5 μg/ml) and the combination of both for 5 days. Results were determined using an MTT assay and are expressed as the percentage of cell proliferation inhibition from three independent experiments performed in triplicate. Columns represent % of cell proliferation inhibition after trastuzumab or pertuzumab exposure and bars SE. * (p ≤ 0.05), ** (p ≤ 0.01) and *** (p ≤ 0.001) indicate levels of statistically significant difference compared with effect of the same drug in SKBr3 cells or compared with drugs administered alone (dashed line).

SK**TR**, SK**LR** and SK**LTR** cells maintained HER2 protein expression levels similar to parental cells (Fig 1B). Therefore, we first checked the effects of the HER2-dimerization inhibitor, pertuzumab, in comparison with trastuzumab in resistant cells. As shown in Fig 2, 5 μg/ml of pertuzumab and 20 μM of trastuzumab were needed to achieve 50% inhibition of cell proliferation (% cpi) of SK parental cells. Same drug concentrations in SK**TR**, SK**LR** and SK**LTR** showed significant resistance to pertuzumab and trastuzumab, compared with SK cells. Pertuzumab only reached 27.8% (*p-value*: *0*.*004*), 38.8% (*p-value*: *0*.*041*) and 19.6% (*p-value*: *0*.*001*) of cpi in SK**TR**, SK**LR** and SK**LTR**, respectively. Similar inhibitory pattern displayed trastuzumab in SK**TR** (19.9%; *p-value*: *0*.*000*), SK**LR** (32.1%; *p-value*: *0*.*022*) and SK**LTR** (15.4%; *p-value*: *0*.*000*) cells.

Co-treatment of resistant cells with trastuzumab *plus* pertuzumab did not increase cytotoxic effect in SK**TR** (25.7% cpi; *p-value*: *0*.*008*), neither in SK**LR** (38.3% cpi; *p-value*: *0*.*023*) cells. But, co-treatments effect in SK and SK**LTR** significantly improved the inhibitory effect up to 63.1% in SK cells (*p-value*: *0*.*007* compared with trastuzumab), and 52.5% in combined treatments (*p-value*_*Pertuzumab*_: *0*.*001* compared with pertuzumab and *p-value*_*Trastuzumab*_: *0*.*000* compared with trastuzumab).

### *In vitro* cell growth interactions between FASN inhibitors and pertuzumab in SK, SKTR, SKLR and SKLTR

Since targeting only HER2 was far from obtaining desired results in resistant models, we decided to explore dual targeting pharmacological strategies. Because FASN showed similar expression levels in parental and resistant cells (Fig 1B), this could be a candidate target to overcome anti-HER2 resistance. Thus, we conducted series of combinatory experiments to evaluate the inhibitory effect of EGCG and G28UCM alone and in combination with pertuzumab in SK**,** SK**TR,** SK**LR** and SK**LTR** cells. The natural anti-FASN compound EGCG had similar cytotoxic effect in parental and resistant cells. IC_**5**0_ values ranged from 206 ± 18.7 μM to 229 ± 29.4 μM in SK, SK**TR**, SK**LR** and SK**LTR** cells. G28UCM, the synthetic derivative of EGCG, improved the cytotoxic effect of EGCG in all cell lines. IC_**5**0_ value of G28UCM in parental and resistant cells ranged from 9 ± 1.5 μM to 19 ± 2.1 μM (S1 Table). In addition and according to our previous results in HER2+ parental cells, EGCG and G28UCM induced apoptosis (cleavage of PARP) in resistant cells (S2 and S3 Figs).

**Fig 3:**
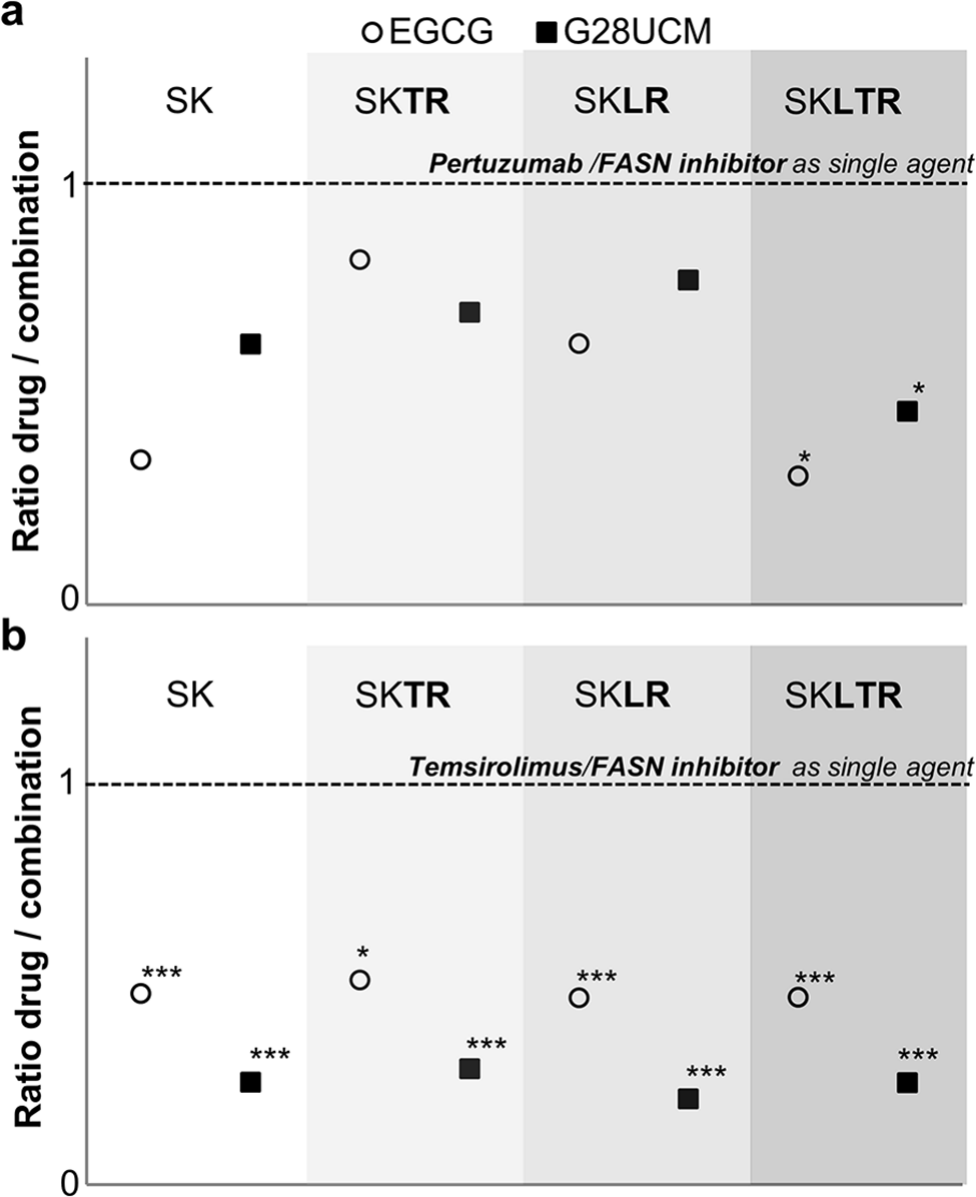
FASN inhibitors improve pertuzumab and temsirolimus activity in parental and resistant cells. **(a)** Cells were treated with pertuzumab (5 μg/ml) combined with EGCG (60 μM) or G28UCM (5 μM) for 5 days. Results were determined using an MTT assay and are expressed as ratio of inhibition of cell proliferation induced for each treatment alone versus inhibition induced for co- treatment from three independent experiments performed in triplicate. Dashed lines represent the effect of each drug alone, ratio 1. **(b)** Cells were treated with temsirolimus (0.05, 0.1, 0.5 and 1 μM) combined with EGCG (60 μM) or G28UCM (5 μM) for 2 days. Results were determined using an MTT assay and are expressed as ratio of inhibition of cell proliferation induced for each treatment alone versus inhibition induced for co- treatment from three independent experiments performed in triplicate and with several temsirolimus concentrations. Dashed lines represent the effect of each drug alone, ratio 1. * (p ≤ 0.05), ** (p ≤ 0.01) and *** (p ≤ 0.001) indicate levels of statistically significant difference compared with drugs administered alone.

Results regarding pertuzumab combinatory experiments with EGCG and G28UCM are shown in Fig 3A. Pertuzumab (5 μg/ml) combined with anti-FASN compounds (60 μM of EGCG or 5 μM of G28UCM) increased cpi in parental and resistant cells. Ratios of cpi induced for treatment**s** alone *versus* inhibition induced for co- treatment was less than 1 in all combinatory experiments. In SK cells, ratio of mono-treatments/combination was 0.34. In SK, SK**LR** and SK**TR**, ratio of mono-treatments/combination was 0.34, 0.82 and 0.62, respectively. In SK**LTR**, monotreatments/combination ratio was 0.31. In this case, pertuzumab *plus* EGCG combination significantly improved effects of each treatment alone (which is graphed as 1 in Fig 3; *p-value*_*compared with 1*_: *0*.*036*). G28UCM *plus* pertuzumab slightly improved EGCG *plus* pertuzumab inhibitory effects in SK**TR** cells (0.69 when combined with G28UCM compared with 0.82 in EGCG case) (Fig 3A).

Together, these data show that the co-exposure of the FASN inhibitors, EGCG and G28UCM, with pertuzumab in parental and resistant HER2+ breast cancer cells is more active than either of the drugs used as a single agent.

### *In vitro* cell growth interactions between FASN inhibitors and temsirolimus in SK, SKTR, SKLR and SKLTR

In our resistant cells we showed changes in EGF family receptors expression and activation without changes in mTOR expression and activation, neither in FASN expression. Since resistant cells express similar levels of mTOR and FASN as same as parental cells, we tested the apoptotic (PARP cleavage) effect of inhibiting mTOR (temsirolimus) and FASN (EGCG) in combination with trastuzumab and/or lapatinib. Temsirolimus or EGCG did not recovered trastuzumab and/or lapatinib sensitivity in resistant cells (S3 Fig). Therefore we conducted experiments to evaluate the inhibitory effect of temsirolimus (mTOR inhibitor) alone and in combination with anti-FASN compounds (EGCG and G28UCM) in the developed resistant HER2+ models (SK**TR,** SK**LR** and SK**LTR**).

Temsirolimus alone displayed a potent anti-proliferative effect in parental and resistant cells. IC_50_ concentration ranged from 9 ± 0.9 μM to 11 ± 0.4 μM in resistant models (S1 Table). Temsirolimus (0.05, 0.1, 0.5 and 1 μM) combined with anti-FASN compounds (60 μM of EGCG or 5 μM of G28UCM) for 2 days increased cpi in parental and resistant cells. Mean ratio**s** of cpi induced for each treatment alone *versus* inhibition induced for co-treatment is shown in Fig 3B EGCG plus temsirolimus ratio was similar in parental and resistant cells (from 0.48 to 0.52 and all significantly different from mono-treatments effect; graphed as 1). When temsirolimus was combined with G28UCM, combinatorial effect was almost doubled (ratios were from 0.22 to 0.30; all *p-values < 0*.*000*).

These results were confirmed by the isobologram method, using a series of isobologram transformations of multiple dose-response curves at an effect level of 30% (IC_30_), an statistical analysis that we have used previously [29]. Simultaneous treatment of SK, SK**TR**, SK**LR** and SK**LTR** cells with EGCG and temsirolimus resulted in a strong synergistic interaction index (0.84 < Ix < 0.94). Combination of G28UCM *plus* temsirolimus had an enhanced synergistic interaction index in parental and resistant cells (0.36 < Ix < 0.58), shown in S1 Table.

Effects of EGCG plus temsirolimus compared with mono-treatments and other combinations (EGCG plus pertuzumab and pertuzumab plus trastuzumab and/or lapatinib) on FASN and mTOR expression were also assessed by western blot analysis (see S4 Fig). No significant inhibition in FASN and mTOR protein levels was seen in any treatment except in the case of EGCG plus temsirolimus combination. EGCG plus temsirolimus completely abolished mTOR expression in SK**TR**, SK**LR** and SK**LTR** resistant cells.

These data show that co-exposure of temsirolimus with FASN inhibitors, EGCG and G28UCM, display a more potent synergistic effect in HER2+ parental and resistant cells than either of the drugs used as a single treatment.

### Antitumor activity of EGCG in combination with pertuzumab and temsirolimus in HER2+ sensitive and resistant patient derived xenografts

**Fig 4:**
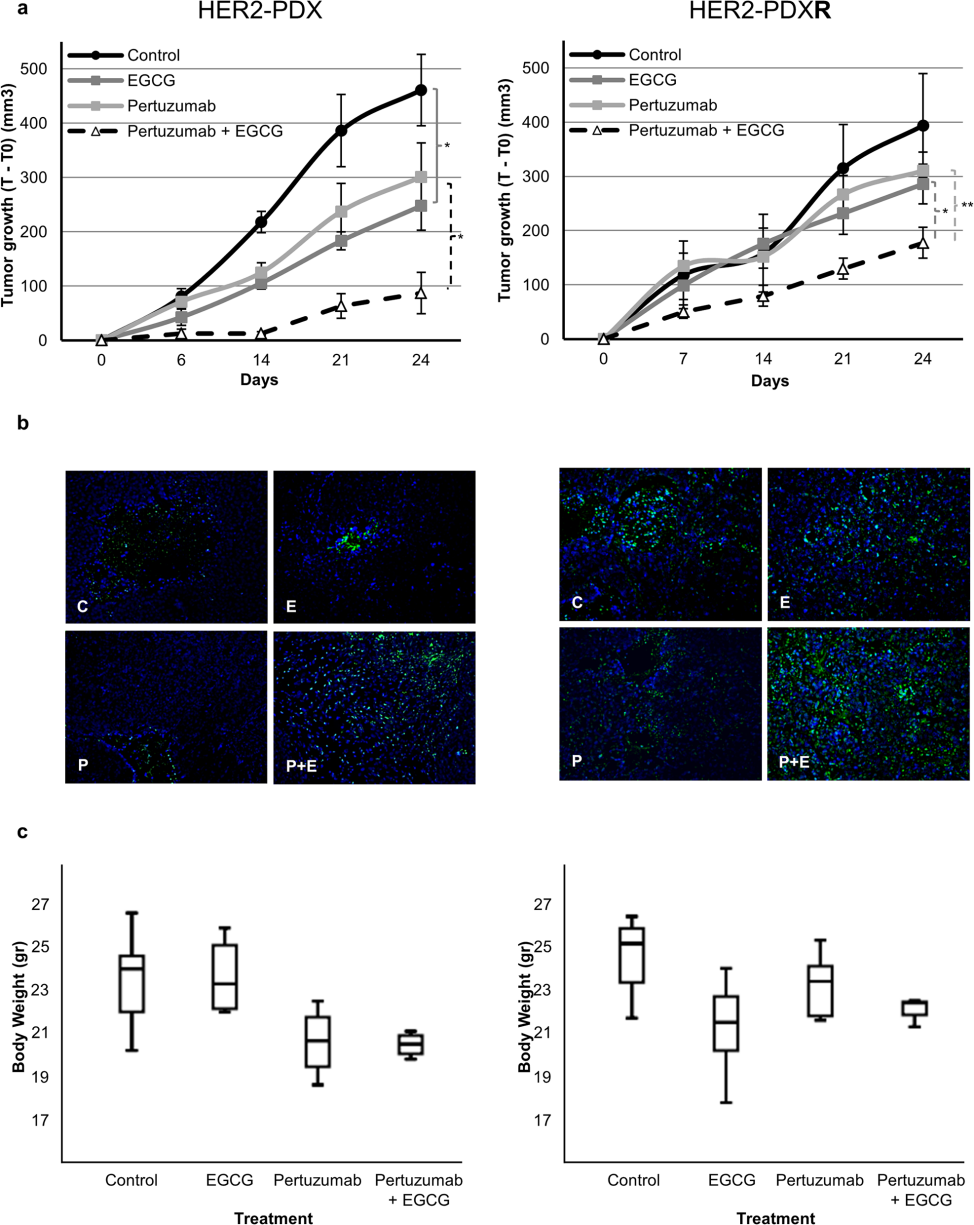
EGCG, alone or combined with pertuzumab, inhibits tumor growth of sensitive and resistant HER2+ orthoxenopatients. **(a)** Mice bearing HER2-PDX and resistant HER2-PDX (HER2-PDXR) were treated with control (C), EGCG (30 mg/kg, 3 days a week), pertuzumab (30 mg/kg, 1 day a week) or the combination (EGCG plus pertuzumab) for 24 days. Dots are mean of each experimental group and bars, SE. * (p ≤ 0.05), ** (p ≤ 0.01) and *** (p ≤ 0.001). **(b)** Apoptosis, by TUNEL fluorescent assay, was perfomed in control (C), EGCG (E), pertuzumab (P) and combination (P+E) treated as in (A) tumors. Tumors were collected at the end of the experiment and fixed in paraffin. Pictures are representative of two samples of each group. **(c)** Body weight of the mice treated as in (A). Data are expressed as body weight at the end of the experiment and boxes show the 25th to 75th percentiles, whereas whiskers extend to the 5th and 95th percentiles.

To better recapitulate the clinical setting we extent our findings *in vivo* evaluating the antitumor activity of pertuzumab, temsirolimus and EGCG and the combination in a HER2+ PDX model (HER2-PDX) and in a trastuzumab *plus* lapatinib-resistant HER2+ PDX (HER2-PDX**R**) model. Both PDX models showed similar HER2, mTOR and FASN expression levels as the *in vitro* parental and resistant cellular models (S5 Fig). EGCG (30 mg/kg for 3d/w) and pertuzumab (30 mg/kg/once weekly), as single agents, reduced tumor growth in the HER2-PDX model after 24 days of treatment. Control animals achieved a median tumor growth of 461.0 ± 65.6 mm^3^ whereas EGCG significantly reduced tumor growth to 247.6 ± 45.0 mm^3^ (*p-value*: *0*.*017)*, and pertuzumab reduced to 301.0 ± 62.9 mm^3^. Interestingly, superior (and more rapid) tumor regression was achieved by dual FASN and HER2 blockade (87.2 ± 38.2 mm^3^; *p-value*_*vsEGCG*_: *0*.*017 and p-value*_*vsPertu*_: *0*.*010*), compared with EGCG or pertuzumab as single agents (Fig 4A, *left panel*). Despite the absence of complete tumor shrinkage, combinatorial treatment significantly reduced tumor growth in the HER2-PDX model. Under the same schedule, in the HER2-PDX**R** model EGCG (30 mg/kg for 3d/w) and pertuzumab (30 mg/kg/once weekly) also reduced tumor growth after 24 days of treatment (Fig 4A, *right panel*), but *in vivo* efficacy of the dual FASN and HER2 blockade was also superior (and more rapid) compared with EGCG and pertuzumab as a single agents. Compared with the control group (393.9 ± 95.5 mm^3^), EGCG and pertuzumab decreased tumor growth to 285.9 ± 36.5 mm^3^ and 310.4 ± 34.5 mm^3^, respectively. The combination of EGCG with pertuzumab significantly reduced tumor growth up to 177.64 ± 34.5 mm^3^ (*p-value*_*vsEGCG*_: *0*.*030 and p-value*_*vsPertu*_: *0*.*008)*.

**Fig 5:**
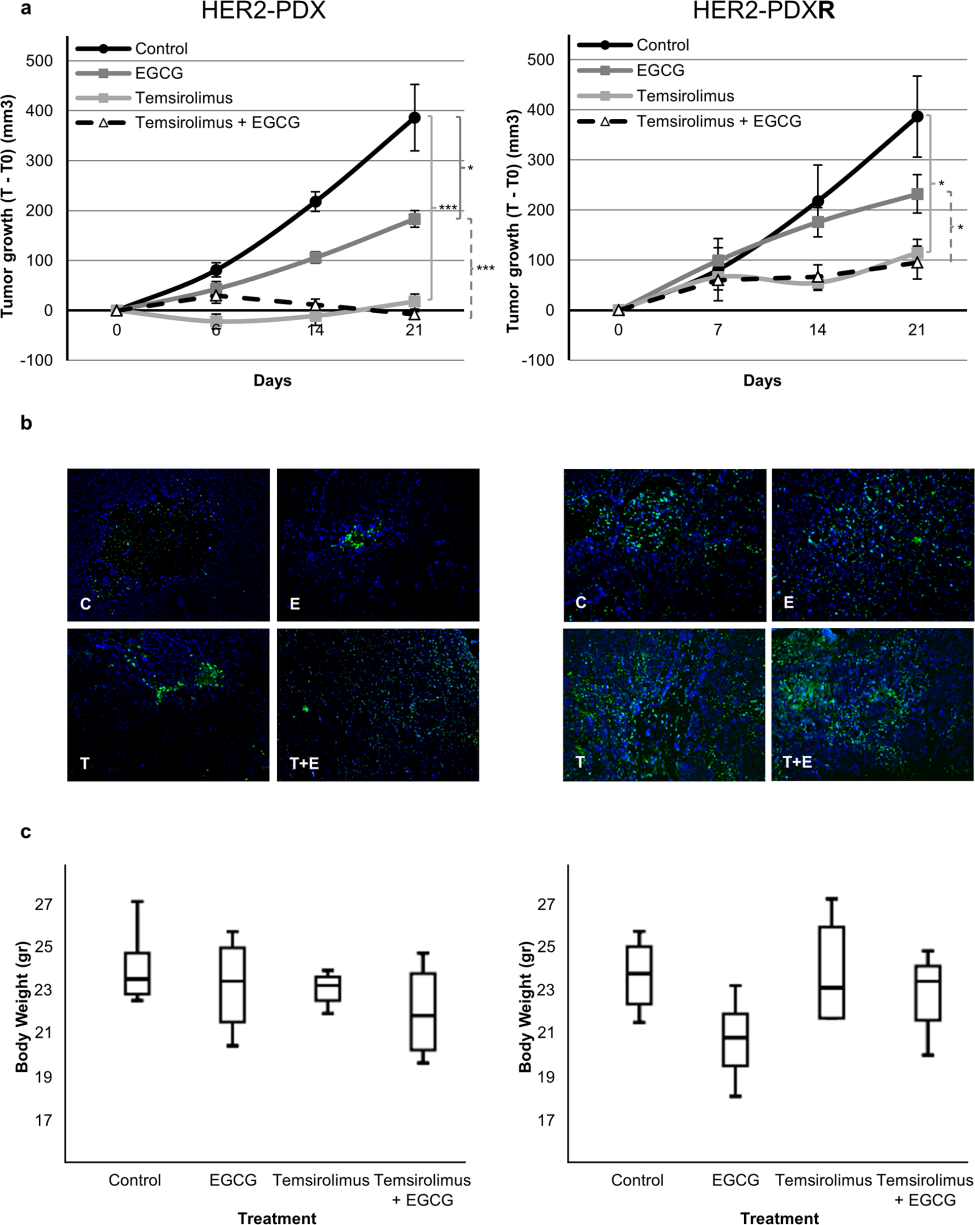
EGCG, alone or combined with temsirolimus, inhibits tumor growth of sensitive and resistant HER2+ orthoxenopatients. **(a)** Mice bearing HER2-PDX and resistant HER2-PDX (HER2-PDXR) were treated with control (C), EGCG (30 mg/kg, 3 days a week), temsirolimus (10 mg/kg, 1 day a week) or the combination (EGCG plus temsirolimus) for 21 days. Dots are mean of each experimental group and bars, SE. * (p ≤ 0.05), ** (p ≤ 0.01) and *** (p ≤ 0.001). **(b)** Apoptosis, by TUNEL fluorescent assay, was perfomed in control (C), EGCG (E), temsirolimus (T) and combination (T+E) treated as in (A) tumors. Tumors were collected at the end of the experiment and fixed in formalin. Pictures are representative of two samples of each group. **(c)** Body weight of the mice treated as in (A). Data are expressed as body weight at the end of the experiment and boxes show the 25th to 75th percentiles, whereas whiskers extend to the 5th and 95th percentiles.

Regarding mTOR and FASN inhibition *in vivo*, EGCG (30 mg/kg for 3d/w) reduced tumor growth in the HER2-PDX model after 21 days of treatment (Fig 5A, *left panel*). Control animals achieved a median tumor growth of 386.4 ± 66.7 mm^3^ whereas EGCG median tumor growth was significantly reduced to 183.3 ± 15.1 mm^3^ (*p-value*: *0*.*017*). Despite the strong antitumor activity exhibited by temsirolimus when used as a single agent (18.0 ± 15.1 mm^3^; *p-value*: *0*.*000*), its activity was little enhanced (day 21) by the addition of EGCG. Combination of temsirolimus with EGCG not only reduced tumor ratio of growth, but also achieved tumor shrinkage compared with the initial tumor volume (-8.2 ± 6.0 mm^3^). In the trastuzumab *plus* lapatinib-resistant HER2-PDX model EGCG treatment decreased the median tumor growth (231.8 ± 38.4 mm^3^) compared with control group (314.8 ± 81.1 mm^3^) at the end of the experiment (Fig 5A, *left panel*). In the HER2-PDX**R** model, temsirolimus significantly decreased tumor growth when used as a single agent (114.3 ± 27.1 mm^3^ *; p-value*: *0*.*045*), and its activity was enhanced by the addition of EGCG (94.9 ± 33.1 mm^3^). These results show that temsirolimus could have a relevant effect in patients with HER2 breast cancer, even those that have progressed to anti-HER2 therapies, and combination with FASN-inhibitors could even assist temsirolimus to achieve tumor depletion.

Tumor samples from HER2-PDX and HER2-PDX**R** treated tumors showed an increased apoptosis compared with HER2-PDX and HER2-PDX**R** control tumors, assessed by fluorescent TUNEL assay (Figs 4B and 5B). EGCG, pertuzumab and temsirolimus used as single agents induced apoptosis in HER2-PDX (133±14 TUNEL+ cells/mm^2^, 122±16 TUNEL+ cells/mm^2^ and 333±19 TUNEL+ cells/mm^2^, respectively) and HER2-PDX**R** (337±19 TUNEL+ cells/mm^2^, 352±18 TUNEL+ cells/mm^2^ and 803±36 TUNEL+ cells/mm^2^, respectively) tumors compared with apoptosis showed by untreated HER2-PDX (66±8 TUNEL+ cells/mm^2^) and HER-PDX**R** (287±23 TUNEL+ cells/mm^2^) tumors. Combinatory treatments (pertuzumab *plus* EGCG and temsirolimus *plus* EGCG) increased the apoptosis in HER2-PDX (933±40 TUNEL+ cells/mm^2^ and 1265±51 TUNEL+ cells/mm^2^, respectively) and HER2-PDX**R** (866±40 TUNEL+ cells/mm^2^ and 1197±55 TUNEL+ cells/mm^2^, respectively) tumors compared with each single treatment alone.

Previous first-generation of FASN inhibitors such as C75 and cerulenin have been limited by inducing severe body weight loss, which is thought to be related to a parallel stimulation of fatty acid oxidation by these inhibitors [24, 37]. But we have previously reported that animals treated with EGCG didn’t display neither change on body weight nor on hepatic, renal and haematological function serum markers compared to control animals [19]. In this study, animals treated with EGCG and also with pertuzumab and temsirolimus (alone or in combination) were weighed daily to evaluate *in vivo* body weight effect. With respect to control animals, we identified no significant changes on food and fluid intake or body weight after treatment with EGCG, pertuzumab and temsirolimus alone or in combination (Figs 4C and 5C).

Histological studies (Hematoxylin-Eosin) of liver, lung, kidney and heart showed no tissue structural abnormalities between control and treated animals in both HER2-PDX models (S6, S7 and S8 Figs).

## Discussion

Despite the remarkable success of anti-HER2 therapies, patients with advanced HER2-positive breast cancer frequently display primary resistance and, in patients initially sensitive to these agents, acquired resistance may emerge over time [9, 10]. To date, even several mechanisms of resistance to anti-HER2 agents are known, this clinical problem is not fully understood. Here, we have developed and characterized stable cell lines derived from the HER2-positive SKBr3 cells that are resistant to either trastuzumab (SK**TR**), lapatinib (SK**LR**) or both (SK**LTR**). Some molecular mechanisms of resistance in our developed anti-HER2 resistant models are consistent with the previously described [8–16]. One of the commonly described mechanisms of anti-HER2 therapies is the overexpression of other RTKs or their ligands. Thus, it has been reported that HER3 overexpression leads to HER2/HER3 heterodimer formation consequently activating the PI3K/AKT/mTOR pathway [6]. Conversely, our trastuzumab, lapatinib and trastuzumab *plus* lapatinib resistant models show a decrease in HER3 expression and activation, whereas an overexpression of EGFR and increased expression levels of EGFR (EGF and TGF-α) and EGFR-HER4 (EREG and HB-EGF) ligands. This is consistent with several reports that show EGFR overexpression (and its ligands) in trastuzumab resistant SKBr3 cells and in xenograft models of acquired trastuzumab and lapatinib resistance [38–40]. Even more, after dual trastuzumab and lapatinib long-term exposure, our patented SK**LTR** [41] cells overexpressed HER4 besides EGFR, and increased the expression of EGFR (EGF and AR) and EGFR-HER4 (BTC, EREG and HB-EGF) ligands to overcome the anticancer effects of both anti-HER2 agents. In a clinical study, constitutive presence of HER4 has been directly related with sensitivity to anti-HER2 drugs in breast cancer [42] whereas in prostate cancer an increase of HER4 expression has been shown to be responsible of resistance to the EGFR inhibitor erlotinib [43]. Our findings highlight that HER4 overexpression and activation could be a new molecular mechanism of resistance to anti-HER2 therapies.

Changes in HER2 downstream proteins (such as loss of PTEN, PI3K mutations/hyperactivation, AKT overexpression and hyperactivation) have also been identified as resistant mechanisms to trastuzumab and lapatinib therapies [14–16]. In patients treated with trastuzumab, activating mutations of PI3K (*PI3KCA*) were associated with poor response and survival [44]. Eichhom PJ *et al* also reported *PI3KCA* mutations as responsible of lapatinib resistance [15]. In this study, we have shown that although *PI3KCA* mutations (PIK3CA_E545K mutation in SK**TR** cells and PIK3CA_E542K mutation in SK**LR** and SKLTR cells) have not enough incidence to show effects in AKT activation, they collaborate with RTKs changes and downstream loops in order to maintain PI3K/AKT/ mTOR pathway activation in trastuzumab, lapatinib and also in a trastuzumab *plus* lapatinib resistant cells. Lapatinib effects have been described to be mediated preferentially through the MAPK/ERK pathway through Ras overexpression or mutation [45]. Accordingly, in our study ERK1/2 overactivation is also shown to be another downstream change that leads to cell proliferation signaling of the resistant cells. No significant changes in mTOR and p-mTOR proteins were observed in our long-term resistant cells. Although the main pathway related to mTOR is the PI3K/AKT axis, mTOR is a downstream in which several signaling pathways converge. In addition, this pathways act as a complex network, and activation of one important effector can be accomplished by several emissaries. Even with decreased activation of AKT in our resistant models, maintenance of mTOR activation can be accomplished by direct signaling of PI3K to mTOR, bypassing AKT [46]. Also, it is described that MAPK inhibit the tuberous sclerosis complex (TSC1/TSC2), which in turn inhibits mTOR activation [47].

Together, overactivation of HER2 in SK**LR** and SK**LTR** cells and maintenance of mTOR, p-mTOR and FASN expression in all the resistant models provided the rationale to test combined FASN and HER2 or mTOR pathways blockade in this setting. We found that the simultaneous treatment of parental and resistant (SK**TR**, SK**LR** and SK**LTR**) cells with anti-FASN compounds (EGCG and the novel derivative G28UCM) *plus* pertuzumab improved the effects of each drug alone in SK**LTR**. But, inhibiting mTOR, the downstream target of HER2 pathway in combination with FASN inhibition resulted in a strong synergistic interaction in all parental and resistant cells. G28UCM, the novel FASN inhibitor, also improved combinatorial effect of EGCG, producing much more synergism between temsirolimus and G28UCM. We had previously shown that G28UCM improved EGCG effects, alone and in several combinatorial regiments with anti-HER2 drugs and chemotherapy, in parental and trastuzumab- or lapatinib-resistant AU565 HER2 breast cancer cells [29]. Several studies have used mTOR inhibition to overcome resistance to HER2-targeted therapies [48] and it has already been assayed in women with trastuzumab-resistance [49]. It has also been shown the synergism between mTOR and FASN inhibition to induce cytotoxicity in ER/HER2-positive breast cancer cell lines [50]. These *in vitro* results support the rationale to test *in vivo* the antitumor efficacy of these agents in a combination regimen in tumors resistant to anti-HER2 therapies.

In previous preclinical studies conducted in nude mice bearing HER2 cells, we and others showed that EGCG displayed *in vivo* antitumor activity without decreasing food intake and induction of weight loss [24, 37]. This is the first study that attempt to evaluate the *in vivo* efficacy and feasibility of dual blockade of FASN and the HER2 signaling pathway in HER2-positive patient tumor samples (HER2-PDX) and in HER2 samples of a patient who relapsed after trastuzumab and lapatinib therapies (HER2-PDX**R**). Here, we report the validity of this approach clearly showing that the combination of EGCG with pertuzumab and temsirolimus resulted in synergistic reduction of HER2-PDX and HER2-PDX**R** tumors, without signs of toxicity (weight loss) related to *in vivo* antitumor efficacy experiments using anti-FASN compounds [24, 28, 29]. Reduction of tumor growth could be accomplished, in part, by an apoptotic event. Increase of apoptosis has been seen in parental and resistant tumor samples treated with EGCG, pertuzumab and temsirolimus. As synergism in tumor reduction after combinatorial treatments, apoptosis has also been synergistically increased when combining EGCG with pertuzumab or temsirolimus. These similar profiles in tumor growth reduction and apoptosis suggest that apoptosis is responsible for tumor growth inhibition. In fact, we had previously shown that EGCG produces apoptosis *in vitro* and *in vivo* [18, 19, 28, 37]. Apoptosis is also consistent with other studies of pertuzumab in cells and mouse models [51, 52]. Temsirolimus has been shown to produce apoptosis in a resistant oropharyngeal carcinoma cell line [53], colorectal cancer cells [54] and other cancers. But little, if any, apoptosis has been seen in different breast cancer cell lines treated with temsirolimus [55]. In this study, we demonstrate that tumor growth inhibition in HER2 breast cancer PDX (non-resistant and resistant) occurs by apoptotic event in tumoral cells, and this is consistent what have been found in other types of cancer. These findings, accordingly with those obtained *in vitro*, encourages us to think that combining FASN inhibitors with temsirolimus or pertuzumab could be one example of a potential combinatorial available in the clinical management of HER2-positive breast cancer patients who progressed to standard treatments.

In this study, we have developed novel mono- and dual- long term trastuzumab *plus* lapatinib resistant breast cancer models to find out new pharmacological strategies to overcome this setting. Then, we have showed *in vitro* and *in vivo* that the inhibition of FASN, alone or in combination with anti-HER2 signaling drugs (temsirolimus and pertuzumab), could have relevant clinical implications in patients who fail to respond to current therapies.

In summary, our findings provide a rationale for the preclinical development of inhibitors of FASN activity in combination with anti-HER2 signaling agents in breast cancer refractory to anti-HER2 therapies.

## Supporting Information

S1 File Additional Materials and Methods. Checking resistance of the developed cells and histological analysis of mice organs.
(DOCX)

S2 File STR analysis of parental and resistant cells. (DOCX)

S3 File Full length Western Blots. (DOCX)

S1 Table Synergy analysis between FASN inhibitors and temsirolimus in parental and resistant cells. Drug cytotoxicity was calculated as the concentration of drug needed to produce 50% of cell death (IC_50_) when parental SKBr3 (SK) or trastuzumab-, lapatinib- and trastuzumab *plus* lapatinib-resistant cells (SK**TR**, SK**LR** and SK**LTR**). Values represent the mean ± SE from at least three independent experiments performed in triplicate. The interaction index (Ix) for temsirolimus plus FASN inhibitors effect was calculated using isobologram analysis. The Ix parameter indicate whether the doses of the two drugs required to produce a given degree of cytotoxicity are greater than (Ix > 1 or antagonism) equal to (Ix = 1 or additivism) or less than (Ix < 1 or synergism) the doses that would be required if the effect of two agents were strictly synergic. Ix mean values ± SE for the two drug treatment were obtained from triplicate studies with different combination treatments and performed at least twice independently. * (*p* < 0.05), ** (*p* < 0.01) and *** (*p* < 0.001) indicate the level of statistical significance of the Ix compared with an Ix of 1.0.
(DOCX)

S1 Fig Checking resistance of the developed resistant cells. **(a)** SKBr3 (SK) parental (ο) and trastuzumab-resistant SKBr3 (SK**TR**, ●) cells where both treated with increasing concentrations of trastuzumab (1–30 μM) for 5 days. **(b)** SKBr3 (SK) parental (with ο) and lapatinib-resistant SKBr3 (SK**LR**, ●) cells where both treated with increasing concentrations of lapatinib (2–30 μM) for 2 days. **c,** Lapatinib-resistant cells (SK**LR**, ●) and trastuzumab plus lapatinib-resistant SKBr3 (SK**LTR**, ●) cells where both treated with 3 μM lapatinib plus increasing concentrations of trastuzumab (1–30 μM) for 5 days. Results are expressed as percentage of surviving cells after drug treatment (mean ± SE), which was determined using an MTT assay. Experiments were performed at least twice in triplicate. * (p < 0.05) and ** (p < 0.01) indicate statistical difference compared with parental cells.
(DOCX)

S2 Fig G28UCM induces apoptosis in parental and resistant cells without affecting FASN expression. Apoptosis and induction of caspase activity were assessed as cleavage of PARP. SKBr3 (SK) parental, trastuzumab-resistant SKBr3 (SK**TR**), lapatinib-resistant SKBr3 (SK**LR**) and lapatinib plus trastuzumab-resistant SKBr3 (SK**LTR**) cells were treated with G28UCM (28 μM) for 24 hours. Control cells were cultured under the same conditions, without treatment for 24 hours. Equal amounts of lysates were immunoblotted with anti-PARP antibody which identified the 116 KDa (intact PARP) and the 89 KDa (cleavage product) bands. Same lysates were also immunobloted with FASN antibody to check G28UCM effect on expression of FASN. Blots were reproved for β-actin as loading control.
(DOCX)

S3 Fig EGCG and temsirolimus improve trastuzumab, lapatinib and trastuzumab plus lapatinib treatment in parental and resistant cells. Apoptosis and induction of caspase activity were assessed as cleavage of PARP. a) SKBr3 (SK) parental cells and b) trastuzumab-resistant SKBr3 (SK**TR**), lapatinib-resistant SKBr3 (SK**LR**) and lapatinib plus trastuzumab-resistant SKBr3 (SK**LTR**) cells were treated with trastuzumab (T; 2 μM), lapatinb (L; 3 μM), EGCG (250 μM) and temsirolimus (Temsi; 12 μM) for 12 and 24 hours. Control cells were cultured under the same conditions, without treatment for 12 or 24 hours. Equal amounts of lysates were immunoblotted with anti-PARP antibody. Blots were reproved for β-actin as loading control.
(DOCX)

S4 Fig Effect of EGCG with pertuzumab and temsirolimus combinations in parental and resistant cells. **a)** SKBr3 (SK) parental cells and **b)** trastuzumab-resistant SKBr3 (SK**TR**), **c)** lapatinib-resistant SKBr3 (SK**LR**) and **d)** lapatinib plus trastuzumab-resistant SKBr3 (SK**LTR**) cells were treated with trastuzumab (T; 2 μM), lapatinb (L; 3 μM), EGCG (250 μM), pertuzumab (5 μg/ml) and temsirolimus (Temsi; 12 μM) for 12 and 24 hours. Control cells were cultured under the same conditions, without treatment for 12 or 24 hours. Equal amounts of lysates were immunoblotted with anti-FASN and anti-mTOR antibodies. Blots were reproved for β-actin as loading control.
(DOCX)

S5 Fig HER2 PDX-tumors characterization. SKBr3 (SK) parental cells and tumors from HER2-PDX and HER2-PDX**R** were lysed and equal amounts of lysates were immunoblotted with anti-HER2, anti-FASN and anti-mTOR antibodies.
(DOCX)

S6 Fig EGCG, alone or combined with pertuzumab, does not induce liver and heart toxicity in xenografts. Histological analysis studies (Hematoxylin-Eosin) of liver and heart showed no tissue structural abnormalities between control and treated animals in both non-resistant and resistant HER2-PDX models. At least 2 mice per group were analyzed and image shown is representative of each group.
(DOCX)

S7 Fig EGCG, alone or combined with temsirolimus, does not induce liver and heart toxicity in xenografts. Histological analysis studies (Hematoxylin-Eosin) of liver and heart showed no tissue structural abnormalities between control and treated animals in both non-resistant and resistant HER2-PDX models. At least 2 mice per group were analyzed and image shown is representative of each group.
(DOCX)

S8 Fig EGCG, alone or combined with pertuzumab and temsirolimus, does not induce kidney and lung toxicity in xenografts. Histological analysis studies (Hematoxylin-Eosin) of kidney and lung showed no tissue structural abnormalities between control and treated animals in both non-resistant and resistant HER2-PDX models. At least 2 mice per group were analysed and image shown is representative of each group.
(DOCX)
