# Supplementary material for: Dual Fatty Acid Synthase and HER2 Signaling Blockade Shows Marked Antitumor Activity against Breast Cancer Models Resistant to Anti-HER2 Drugs
Source: PLoS One. 2015 Jun 24;10(6):e0131241. doi: 10.1371/journal.pone.0131241 (PMC4479882; doi:10.1371/journal.pone.0131241)
Supplement: S1 File — Checking resistance of the developed cells and histological analysis of mice organs. (DOCX) [file pone.0131241.s001.docx]

**File S1. Additional Materials and Methods.** Checking resistance of the developed cells and histological analysis of mice organs.

**Checking the resistance of the developed cells**

SK, SK**TR**, SK**LR**, SK**LTR** were plated out at density of 4 x 10^3^ cells/100 µL/well in 96 well microtitre plates. After overnight cell adherence, the medium was removed and fresh medium along with the corresponding concentrations of trastuzumab (1-30 µM) or lapatinib (2-30 µM) or 3 µM of lapatinib (concentration at which SK**LR** and SK**LTR** are resistant) *plus* increasing concentrations of trastuzumab (1-30 µM) were added to the microtitre culture plates. Drug treatment was not renewed during the period of cell exposure (2 days for lapatinib, 3 days for trastuzumab and 5 days for trastuzumab *plus* lapatinib), and control cells without treatment were cultured under the same conditions with comparable media changes. Following treatment, the media was replaced by drug-free medium (100 μL/well) containing 3,4,5-dimethylthiazol-2-yl-2,5-diphenyltetrazolium bromide (MTT) solution (10 μL, 5 mg/mL in PBS) (MTT) (Sigma), and incubation was prolonged for 3 h at 37 ºC. After carefully removing the supernatants, the MTT-formazan crystals formed by metabolically viable cells were dissolved in DMSO (100 μL/well) and absorbance was determined at 570 nm in a multi-well plate reader (Spectra max 340PC (380), BioNova Cientifica S.L., Madrid, Spain). Using control optical density OD values the trend line was obtained by the formula (ODCTRL- ODTEST)*100/ODCTRL. The data presented are from three separate wells per assay and the assay was performed at least three times.

**Histological analysis**

A minimum of two mice per treatment- or control-group were included in histological analysis. For each mouse, heart and liver were fixed in formalin and included in paraffin blocks. Ten-micron-thick parallel paraffin sections were obtained with a microtome (Leica Microtome RM2235), and mounted on Super Frost slides (Super frost Plus, Thermo). Random representative slices of each organ were labeled with Hematoxyline-Eosine. After washing, slides were dehydrated in alcohol, cleared in xylene and cover slipped with DPX (44581, Fluka). In all treatments and organs of both groups, a minimum of three slices for each labeling were analyzed. These slides were used for microscopic study of possible structural alterations. Two researchers were blinded to sample identity when microscopic analysis was performed. Anatomical landmarks were used to ensure that parameters were analyzed at similar levels within and between samples.
