# Supplementary figures and images for: Dual Fatty Acid Synthase and HER2 Signaling Blockade Shows Marked Antitumor Activity against Breast Cancer Models Resistant to Anti-HER2 Drugs
Source: PLoS One. 2015 Jun 24;10(6):e0131241. doi: 10.1371/journal.pone.0131241 (PMC4479882; doi:10.1371/journal.pone.0131241)

**File S2. STR analysis of parental and resistant cells.**


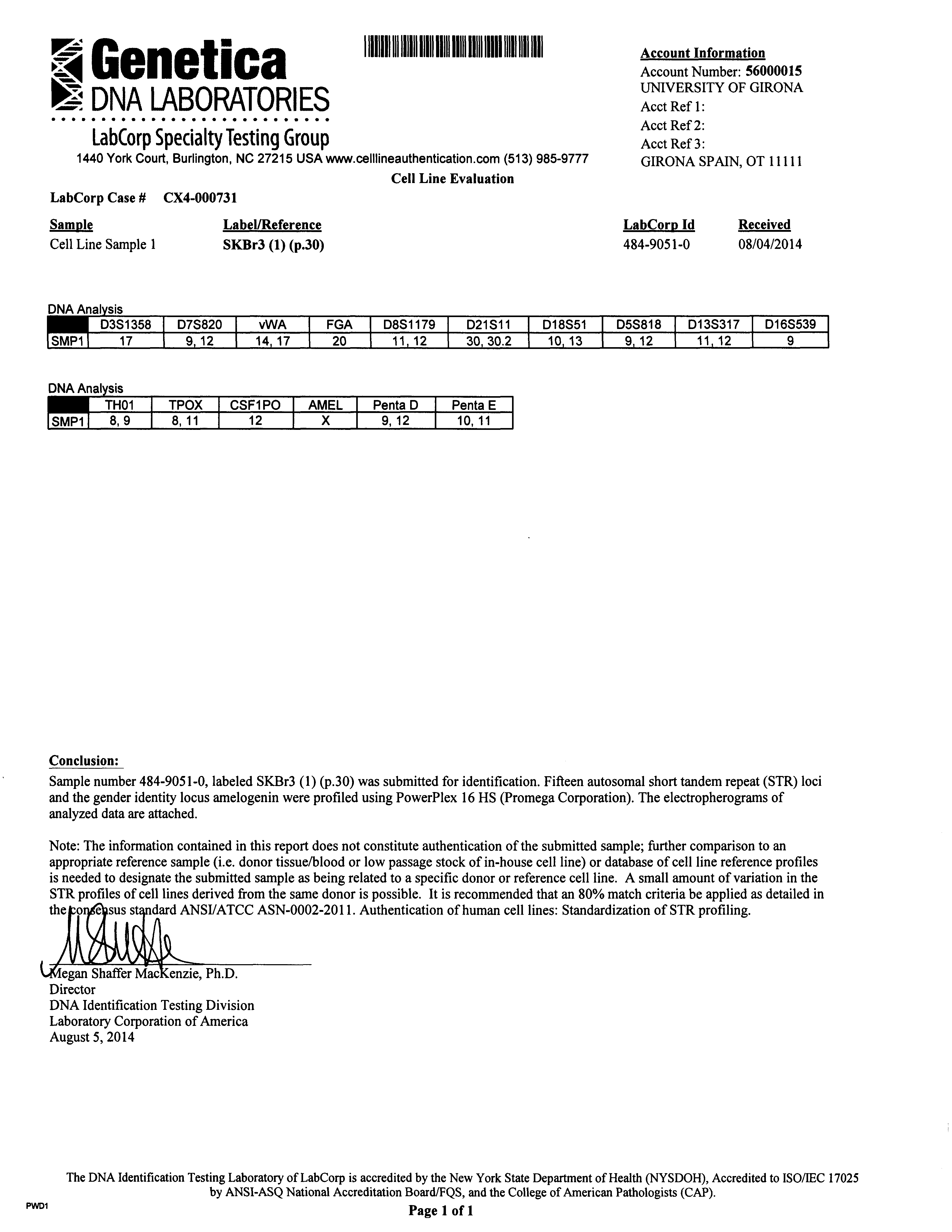


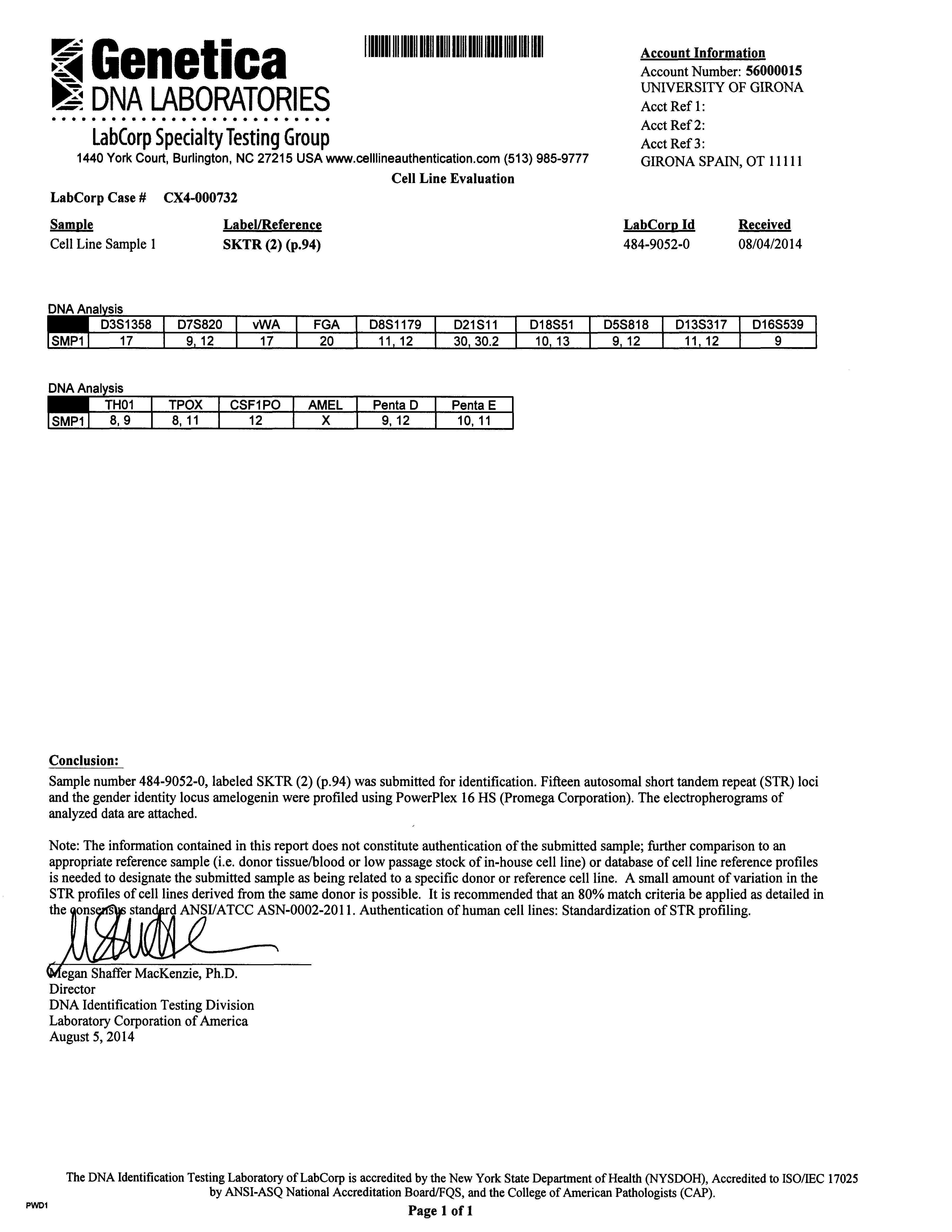


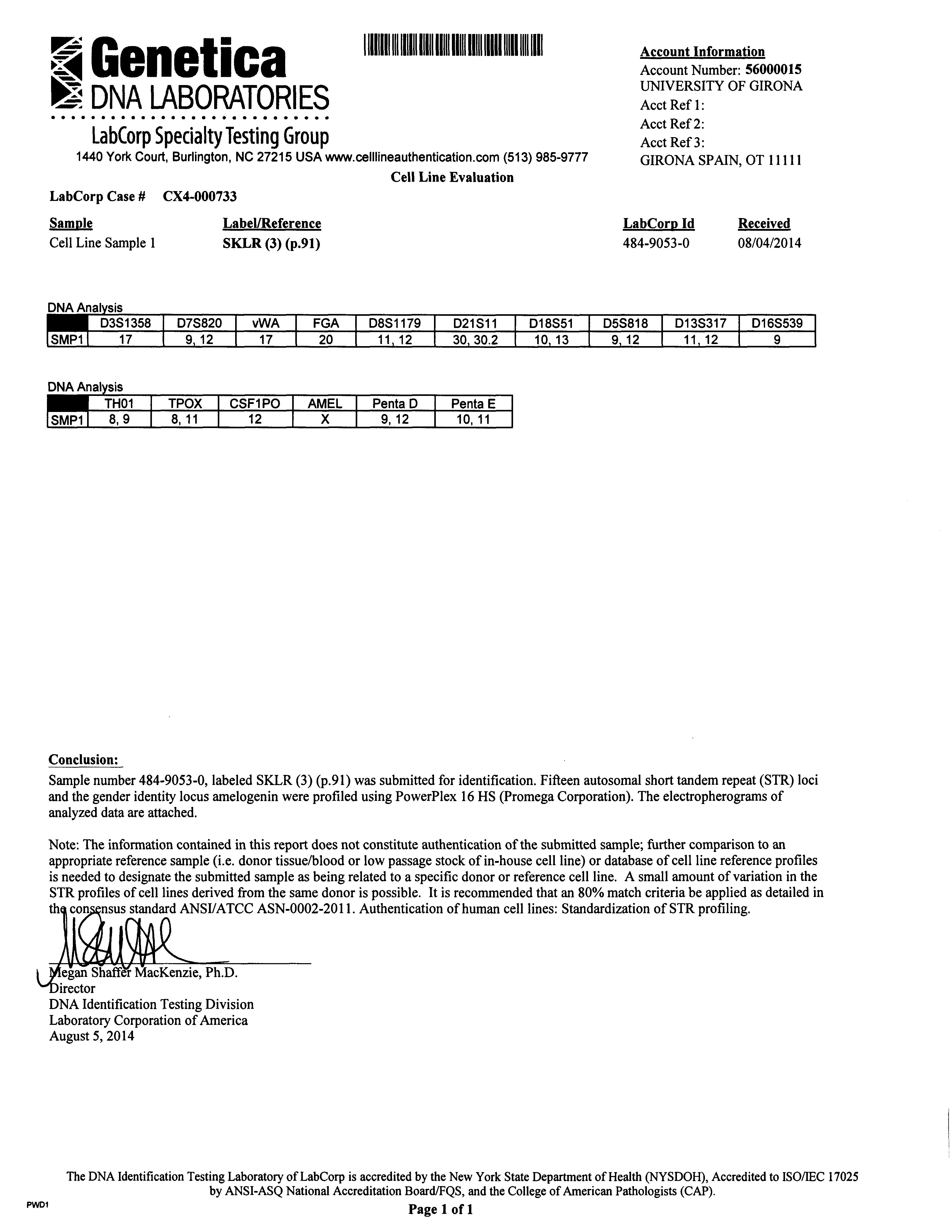


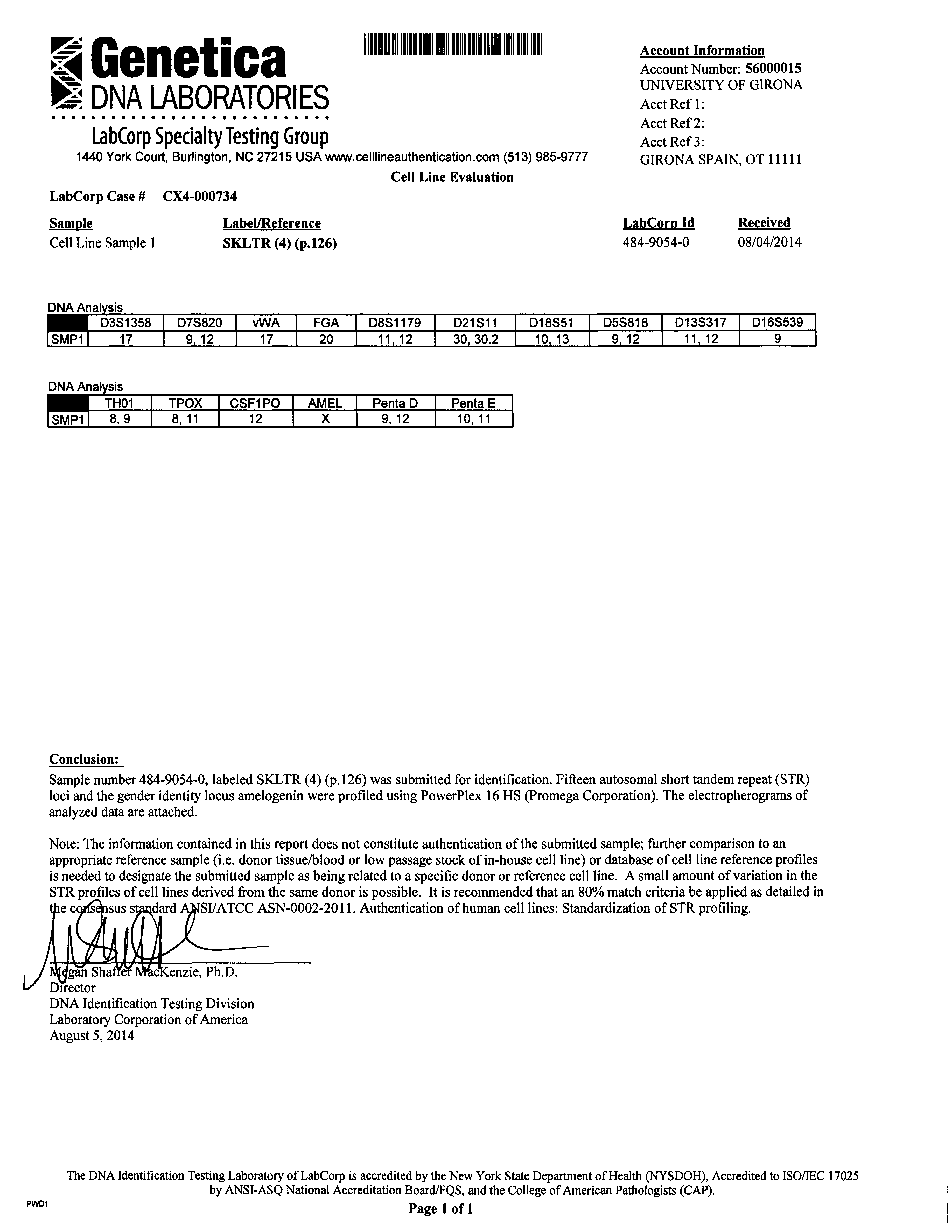

Supplement: S2 File — (DOCX) [file pone.0131241.s002.docx]

**File S3. Full length Western Blots.**

*
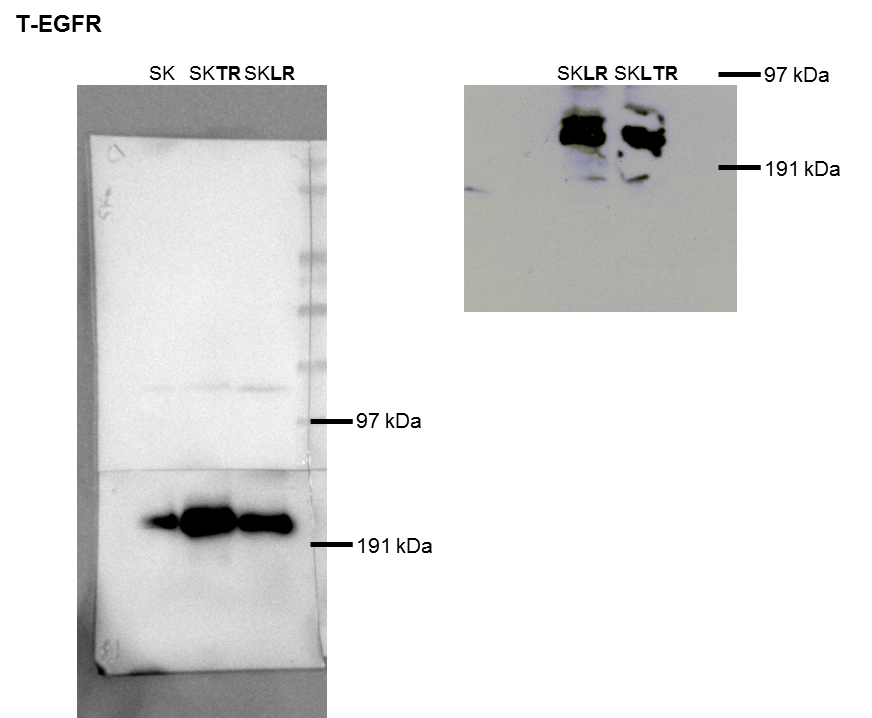
*

*
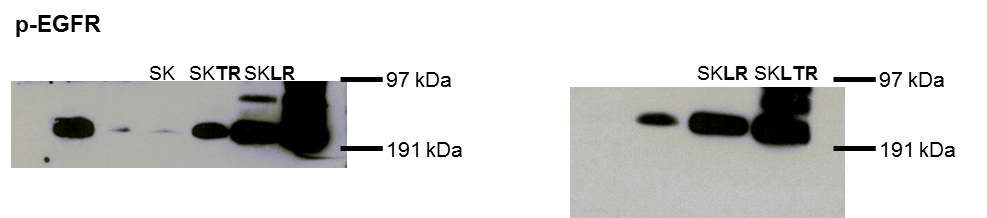
*

*
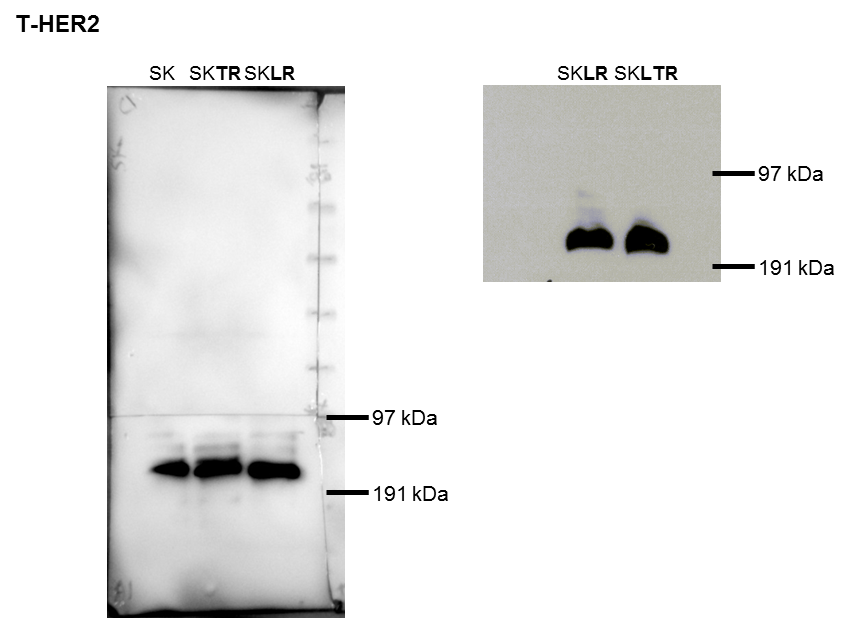
*

*
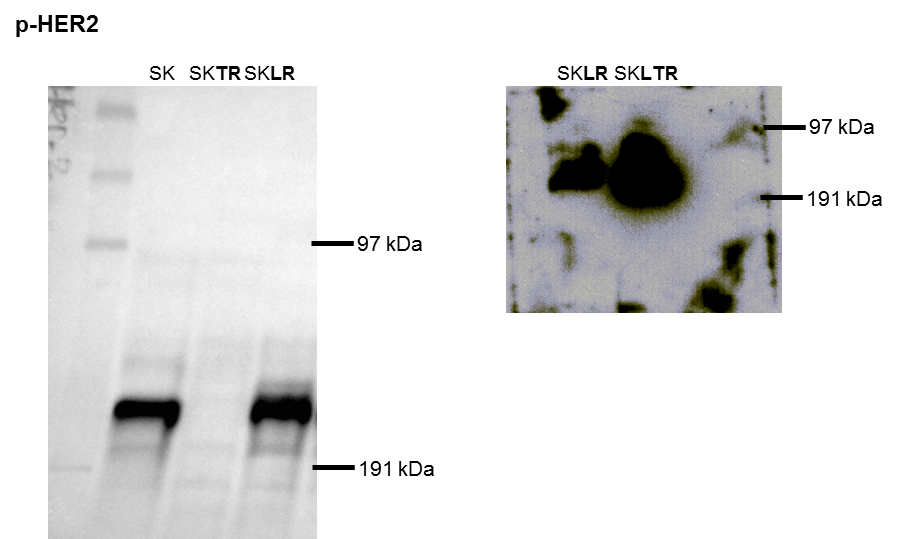
*

*
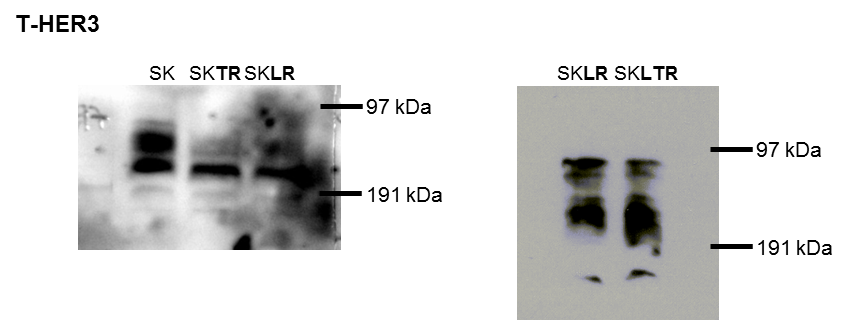
*

*
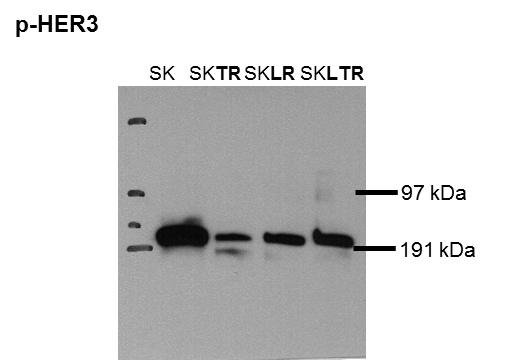
*

*
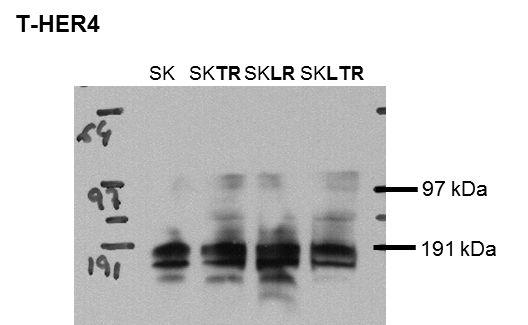
*

*
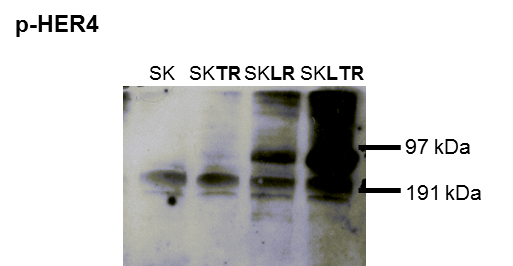
*

*
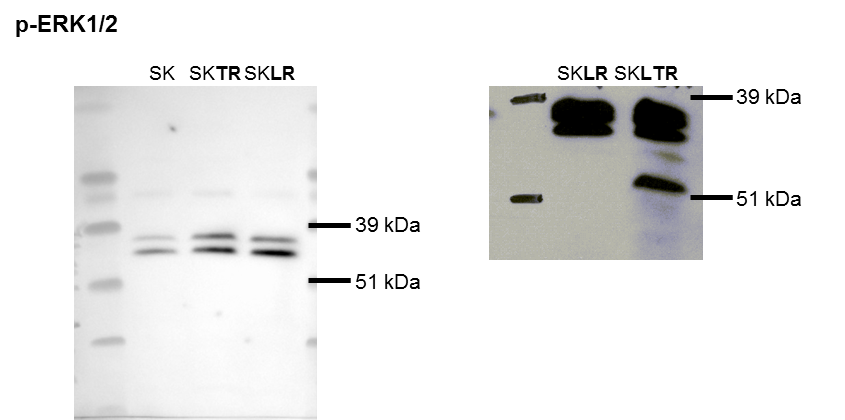

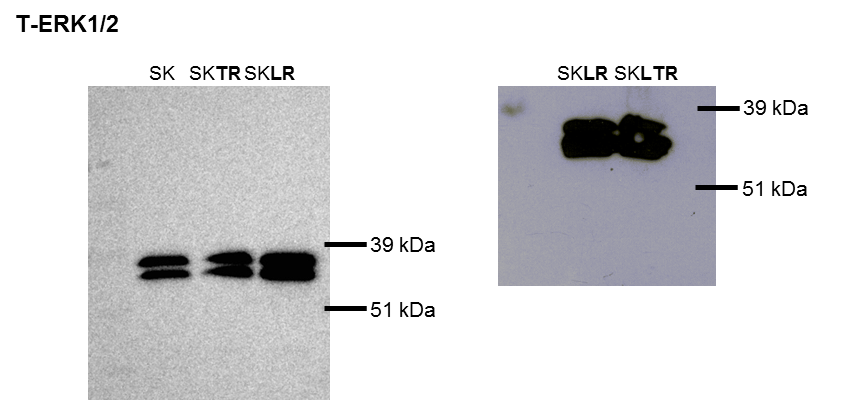
*

*
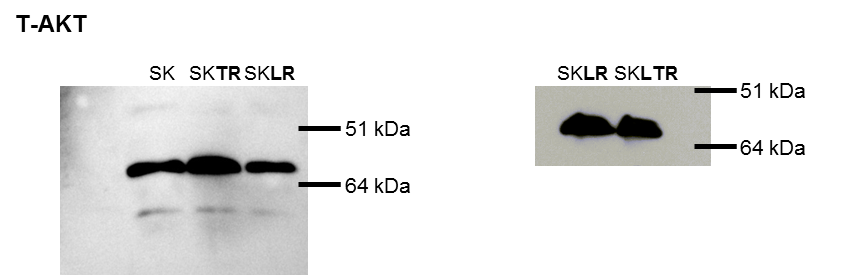
*

*
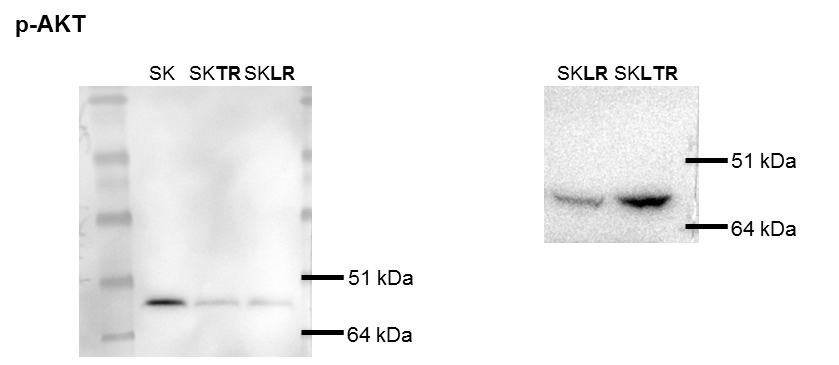
*

*
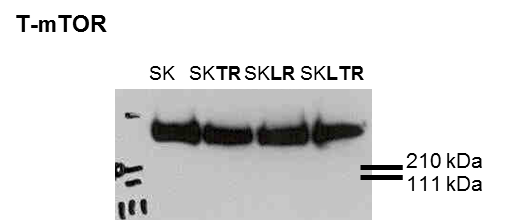
*

*
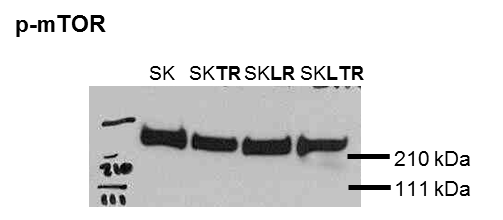
*

*
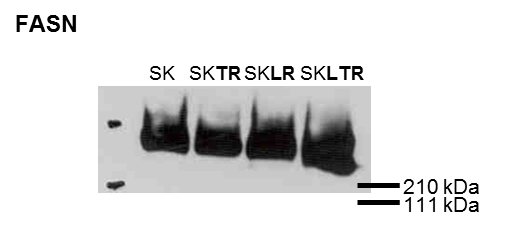
*

*
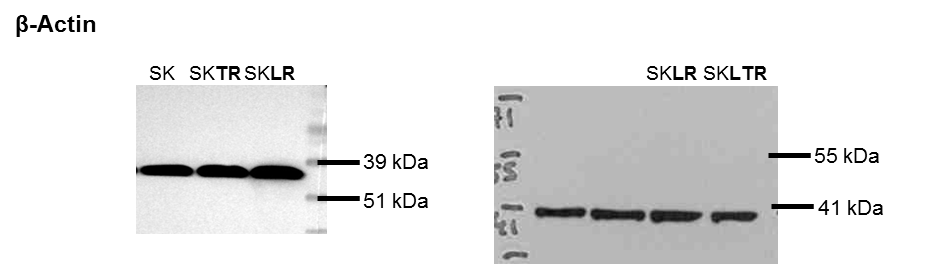
*

Supplement: S3 File — (DOCX) [file pone.0131241.s003.docx]
