# Supplementary material for: Dual Fatty Acid Synthase and HER2 Signaling Blockade Shows Marked Antitumor Activity against Breast Cancer Models Resistant to Anti-HER2 Drugs
Source: PLoS One. 2015 Jun 24;10(6):e0131241. doi: 10.1371/journal.pone.0131241 (PMC4479882; doi:10.1371/journal.pone.0131241)
Supplement: S1 Table — Drug cytotoxicity was calculated as the concentration of drug needed to produce 50% of cell death (IC50) when parental SKBr3 (SK) or trastuzumab-, lapatinib- and trastuzumab plus lapatinib-resistant cells (SKTR, SKLR and SKLTR). Values represent the mean ± SE from at least three independent experiments performed in triplicate. The interaction index (Ix) for temsirolimus plus FASN inhibitors effect was calculated using isobologram analysis. The Ix parameter indicate whether the doses of the two drugs required to produce a given degree of cytotoxicity are greater than (Ix > 1 or antagonism) equal to (Ix = 1 or additivism) or less than (Ix < 1 or synergism) the doses that would be required if the effect of two agents were strictly synergic. Ix mean values ± SE for the two drug treatment were obtained from triplicate studies with different combination treatments and performed at least twice independently. * (p < 0.05), ** (p < 0.01) and *** (p < 0.001) indicate the level of statistical significance of the Ix compared with an Ix of 1.0. (DOCX) [file pone.0131241.s004.docx]

**Table S1. Synergy analysis between FASN inhibitors and temsirolimus in parental and resistant cells.**

|  | SK | SK**TR** | SK**LR** | SK**LTR** |
| --- | --- | --- | --- | --- |
|  | IC_50_ (μM) | IC_50_ (μM) | IC_50_ (μM) | IC_50_ (μM) |
| *Temsirolimus* | 9 ± 0.9 | 11 ± 0.4 | 10 ± 0.5 | 10 ± 1.0 |
| *EGCG* | 211 ± 8.0 | 229 ±29.4 | 206 ± 18.7 | 211 ± 6.7 |
| *G28UCM* | 19 ± 2.1 | 16 ± 1.8 | 17 ± 1.5 | 9 ± 1.5 |
| ***Temsirolimus***  ***+ EGCG*** | *Synergism*  Ix = 0.89 ± 0.01*** | *Synergism*  Ix = 0.84 ± 0.01** | *Synergism*  Ix = 0.88 ± 0.01*** | *Synergism*  Ix = 0.94 ± 0.01** |
| ***Temsirolimus***  ***+ G28UCM*** | *Synergism*  Ix = 0.41 ± 0.03** | *Synergism*  Ix = 0.58 ± 0.02** | *Synergism*  Ix = 0.36 ± 0.03*** | *Synergism*  Ix = 0.57 ± 0.04** |

Drug cytotoxicity was calculated as the concentration of drug needed to produce 50% of cell death (IC_50_) when parental SKBr3 (SK) or trastuzumab-, lapatinib- and trastuzumab *plus* lapatinib-resistant cells (SK**TR**, SK**LR** and SK**LTR**). Values represent the mean ± SE from at least three independent experiments performed in triplicate. The interaction index (Ix) for temsirolimus plus FASN inhibitors effect was calculated using isobologram analysis. The Ix parameter indicate whether the doses of the two drugs required to produce a given degree of cytotoxicity are greater than (Ix > 1 or antagonism) equal to (Ix = 1 or additivism) or less than (Ix < 1 or synergism) the doses that would be required if the effect of two agents were strictly synergic. Ix mean values ± SE for the two drug treatment were obtained from triplicate studies with different combination treatments and performed at least twice independently. * (*p* < 0.05), ** (*p* < 0.01) and *** (*p* < 0.001) indicate the level of statistical significance of the Ix compared with an Ix of 1.0.
