# Supplementary material for: Dual Fatty Acid Synthase and HER2 Signaling Blockade Shows Marked Antitumor Activity against Breast Cancer Models Resistant to Anti-HER2 Drugs
Source: PLoS One. 2015 Jun 24;10(6):e0131241. doi: 10.1371/journal.pone.0131241 (PMC4479882; doi:10.1371/journal.pone.0131241)
Supplement: S1 Fig — (a) SKBr3 (SK) parental (ο) and trastuzumab-resistant SKBr3 (SKTR, ●) cells where both treated with increasing concentrations of trastuzumab (1–30 μM) for 5 days. (b) SKBr3 (SK) parental (with ο) and lapatinib-resistant SKBr3 (SKLR, ●) cells where both treated with increasing concentrations of lapatinib (2–30 μM) for 2 days. c, Lapatinib-resistant cells (SKLR, ●) and trastuzumab plus lapatinib-resistant SKBr3 (SKLTR, ●) cells where both treated with 3 μM lapatinib plus increasing concentrations of trastuzumab (1–30 μM) for 5 days. Results are expressed as percentage of surviving cells after drug treatment (mean ± SE), which was determined using an MTT assay. Experiments were performed at least twice in triplicate. * (p < 0.05) and ** (p < 0.01) indicate statistical difference compared with parental cells. (DOCX) [file pone.0131241.s005.docx]

**Figure S1. Checking the resistance of the developed resistant cells.**

**a,** SKBr3 (SK) parental (ο) and trastuzumab-resistant SKBr3 (SK**TR**, ●) cells where both treated with increasing concentrations of trastuzumab (1 – 30 μM) for 5 days. **b,** SKBr3 (SK) parental (with ο) and lapatinib-resistant SKBr3 (SK**LR**, ●) cells where both treated with increasing concentrations of lapatinib (2 – 30 μM) for 2 days. **c,** Lapatinib-resistant cells (SK**LR**, ●) and trastuzumab *plus* lapatinib-resistant SKBr3 (SK**LTR**, ●) cells where both treated with 3 μM lapatinib *plus* increasing concentrations of trastuzumab (1 – 30 μM) for 5 days. Results are expressed as percentage of surviving cells after drug treatment (mean ± SE), which was determined using an MTT assay. Experiments were performed at least twice in triplicate. * (p < 0.05) and ** (p < 0.01) indicate statistical difference compared with parental cells.


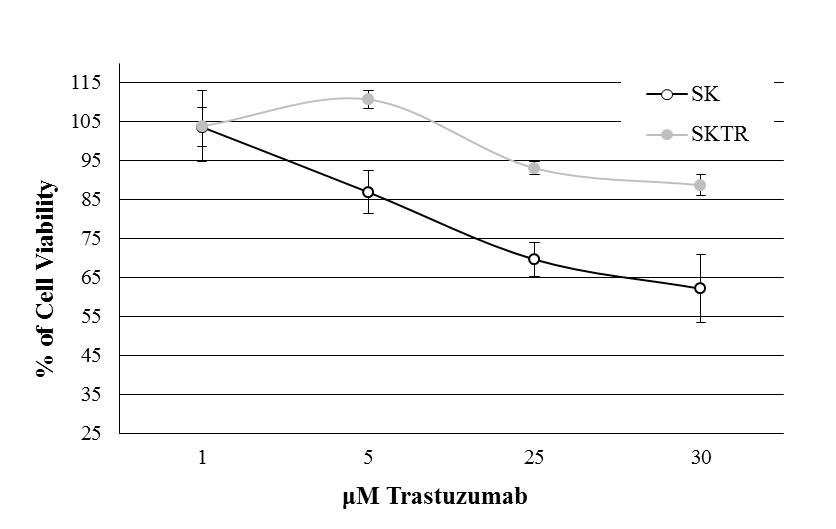


*

*

SK

SK**TR**

**a**


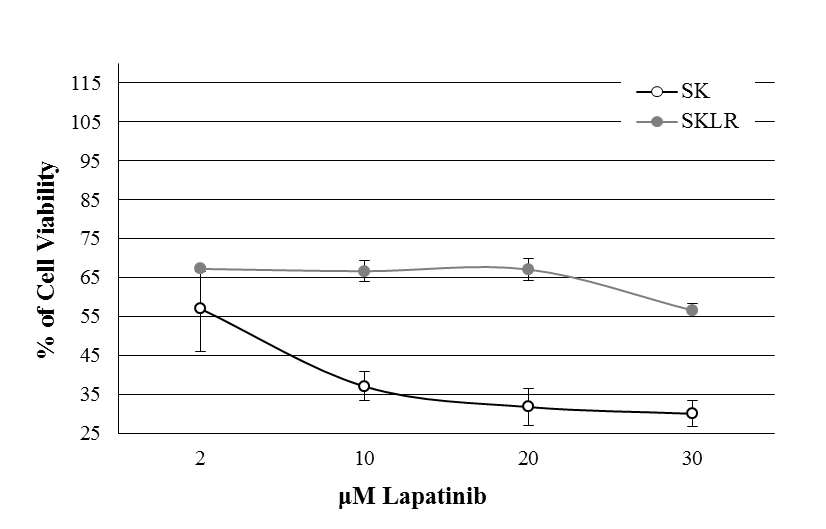


**

**

*

SK

SK**LR**

**b**


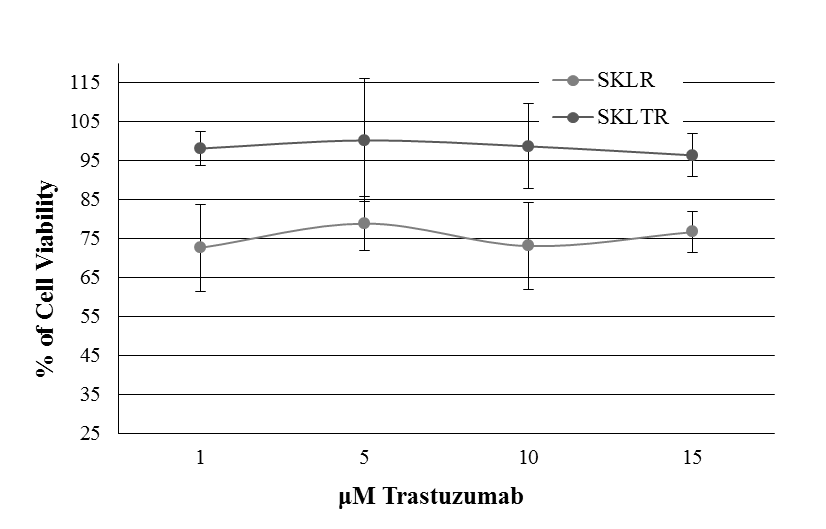


*

**

**

SK**LR**

SK**LTR**

**c**
