# Supplementary material for: Dual Fatty Acid Synthase and HER2 Signaling Blockade Shows Marked Antitumor Activity against Breast Cancer Models Resistant to Anti-HER2 Drugs
Source: PLoS One. 2015 Jun 24;10(6):e0131241. doi: 10.1371/journal.pone.0131241 (PMC4479882; doi:10.1371/journal.pone.0131241)
Supplement: S2 Fig — Apoptosis and induction of caspase activity were assessed as cleavage of PARP. SKBr3 (SK) parental, trastuzumab-resistant SKBr3 (SKTR), lapatinib-resistant SKBr3 (SKLR) and lapatinib plus trastuzumab-resistant SKBr3 (SKLTR) cells were treated with G28UCM (28 μM) for 24 hours. Control cells were cultured under the same conditions, without treatment for 24 hours. Equal amounts of lysates were immunoblotted with anti-PARP antibody which identified the 116 KDa (intact PARP) and the 89 KDa (cleavage product) bands. Same lysates were also immunobloted with FASN antibody to check G28UCM effect on expression of FASN. Blots were reproved for β-actin as loading control. (DOCX) [file pone.0131241.s006.docx]

**Figure S2. G28UCM induces apoptosis in parental and resistant cells without affecting FASN expression.**


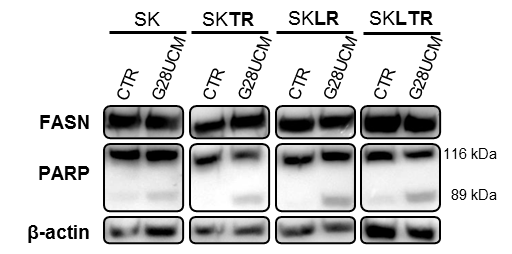
Apoptosis and induction of caspase activity were assessed as cleavage of PARP. SKBr3 (SK) parental, trastuzumab-resistant SKBr3 (SK**TR**), lapatinib-resistant SKBr3 (SK**LR**) and lapatinib plus trastuzumab-resistant SKBr3 (SK**LTR**) cells were treated with G28UCM (28 μM) for 24 hours. Control cells were cultured under the same conditions, without treatment for 24 hours. Equal amounts of lysates were immunoblotted with anti-PARP antibody which identified the 116 KDa (intact PARP) and the 89 KDa (cleavage product) bands. Same lysates were also immunobloted with FASN antibody to check G28UCM effect on expression of FASN. Blots were reproved for β-actin as loading control.
