# Supplementary material for: Dual Fatty Acid Synthase and HER2 Signaling Blockade Shows Marked Antitumor Activity against Breast Cancer Models Resistant to Anti-HER2 Drugs
Source: PLoS One. 2015 Jun 24;10(6):e0131241. doi: 10.1371/journal.pone.0131241 (PMC4479882; doi:10.1371/journal.pone.0131241)

**Figure S4. Effect of EGCG with pertuzumab and temsirolimus combinations in parental and resistant cells.**

**a)** SKBr3 (SK) parental cells and **b)** trastuzumab-resistant SKBr3 (SK**TR**), **c)** lapatinib-resistant SKBr3 (SK**LR**) and **d)** lapatinib plus trastuzumab-resistant SKBr3 (SK**LTR**) cells were treated with trastuzumab (T; 2 µM), lapatinb (L; 3 µM), EGCG (250 µM), pertuzumab (5 µg/ml) and temsirolimus (Temsi; 12 µM) for 12 and 24 hours. Control cells were cultured under the same conditions, without treatment for 12 or 24 hours. Equal amounts of lysates were immunoblotted with anti-FASN and anti-mTOR antibodies. Blots were reproved for β-actin as loading control.


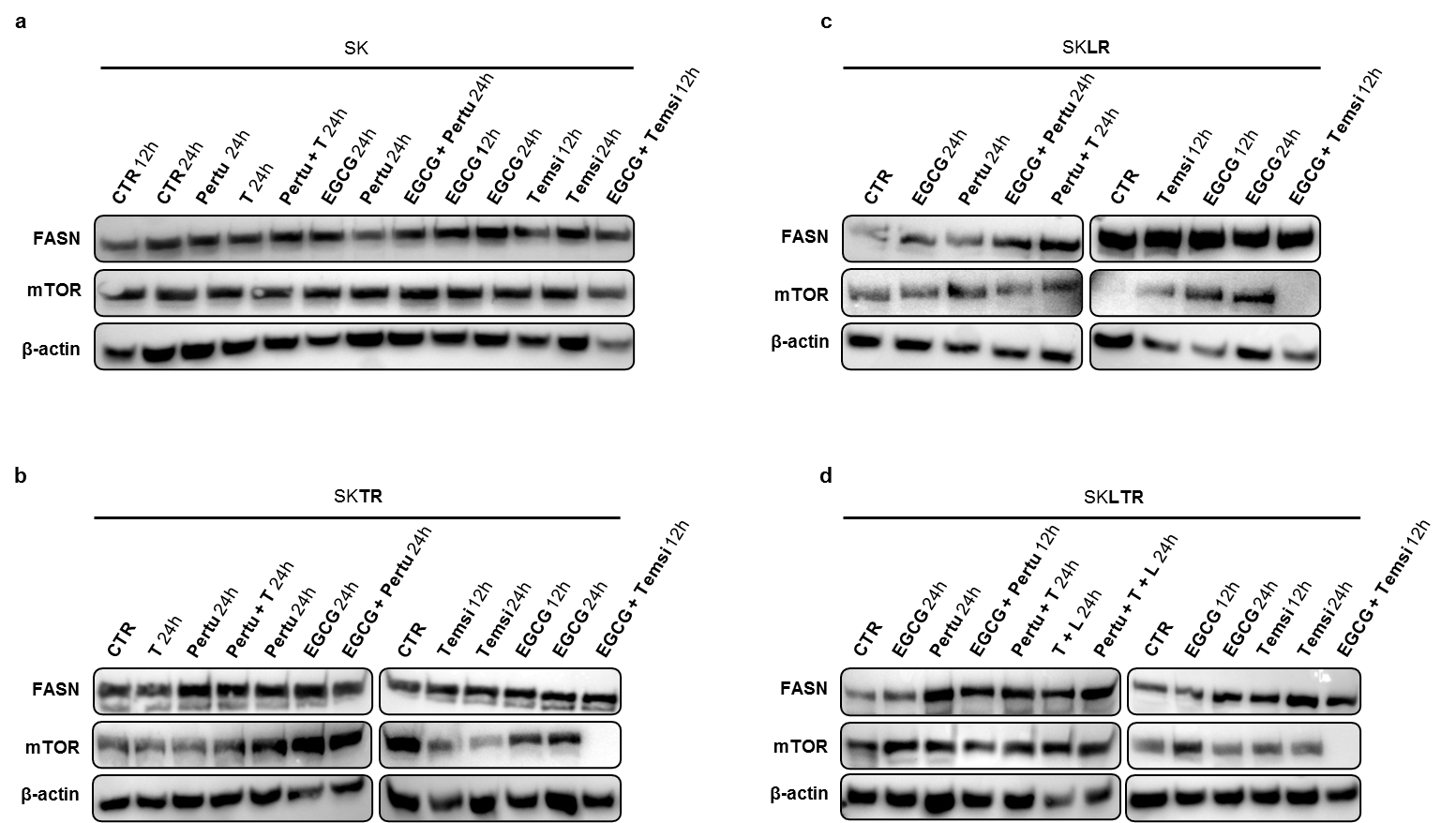

Supplement: S4 Fig — a) SKBr3 (SK) parental cells and b) trastuzumab-resistant SKBr3 (SKTR), c) lapatinib-resistant SKBr3 (SKLR) and d) lapatinib plus trastuzumab-resistant SKBr3 (SKLTR) cells were treated with trastuzumab (T; 2 μM), lapatinb (L; 3 μM), EGCG (250 μM), pertuzumab (5 μg/ml) and temsirolimus (Temsi; 12 μM) for 12 and 24 hours. Control cells were cultured under the same conditions, without treatment for 12 or 24 hours. Equal amounts of lysates were immunoblotted with anti-FASN and anti-mTOR antibodies. Blots were reproved for β-actin as loading control. (DOCX) [file pone.0131241.s008.docx]
