# Supplementary material for: Dual Fatty Acid Synthase and HER2 Signaling Blockade Shows Marked Antitumor Activity against Breast Cancer Models Resistant to Anti-HER2 Drugs
Source: PLoS One. 2015 Jun 24;10(6):e0131241. doi: 10.1371/journal.pone.0131241 (PMC4479882; doi:10.1371/journal.pone.0131241)

**Figure S5. HER2 PDX-tumors characterization.**

SKBr3 (SK) parental cells and tumors from HER2-PDX and HER2-PDX**R** were lysed and equal amounts of lysates were immunoblotted with anti-HER2, anti-FASN and anti-mTOR antibodies.


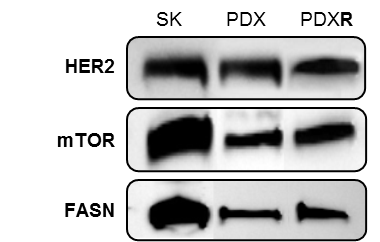

Supplement: S5 Fig — SKBr3 (SK) parental cells and tumors from HER2-PDX and HER2-PDXR were lysed and equal amounts of lysates were immunoblotted with anti-HER2, anti-FASN and anti-mTOR antibodies. (DOCX) [file pone.0131241.s009.docx]
