# Supplementary material for: Dual Fatty Acid Synthase and HER2 Signaling Blockade Shows Marked Antitumor Activity against Breast Cancer Models Resistant to Anti-HER2 Drugs
Source: PLoS One. 2015 Jun 24;10(6):e0131241. doi: 10.1371/journal.pone.0131241 (PMC4479882; doi:10.1371/journal.pone.0131241)
Supplement: S7 Fig — Histological analysis studies (Hematoxylin-Eosin) of liver and heart showed no tissue structural abnormalities between control and treated animals in both non-resistant and resistant HER2-PDX models. At least 2 mice per group were analyzed and image shown is representative of each group. (DOCX) [file pone.0131241.s011.docx]

**Figure S7. EGCG, alone or combined with temsirolimus, does not induce liver and heart toxicity in xenografts.**

Histological analysis studies (Hematoxylin-Eosin) of liver and heart showed no tissue structural abnormalities between control and treated animals in both non-resistant and resistant HER2-PDX models. At least 2 mice per group were analyzed and image shown is representative of each group.

**
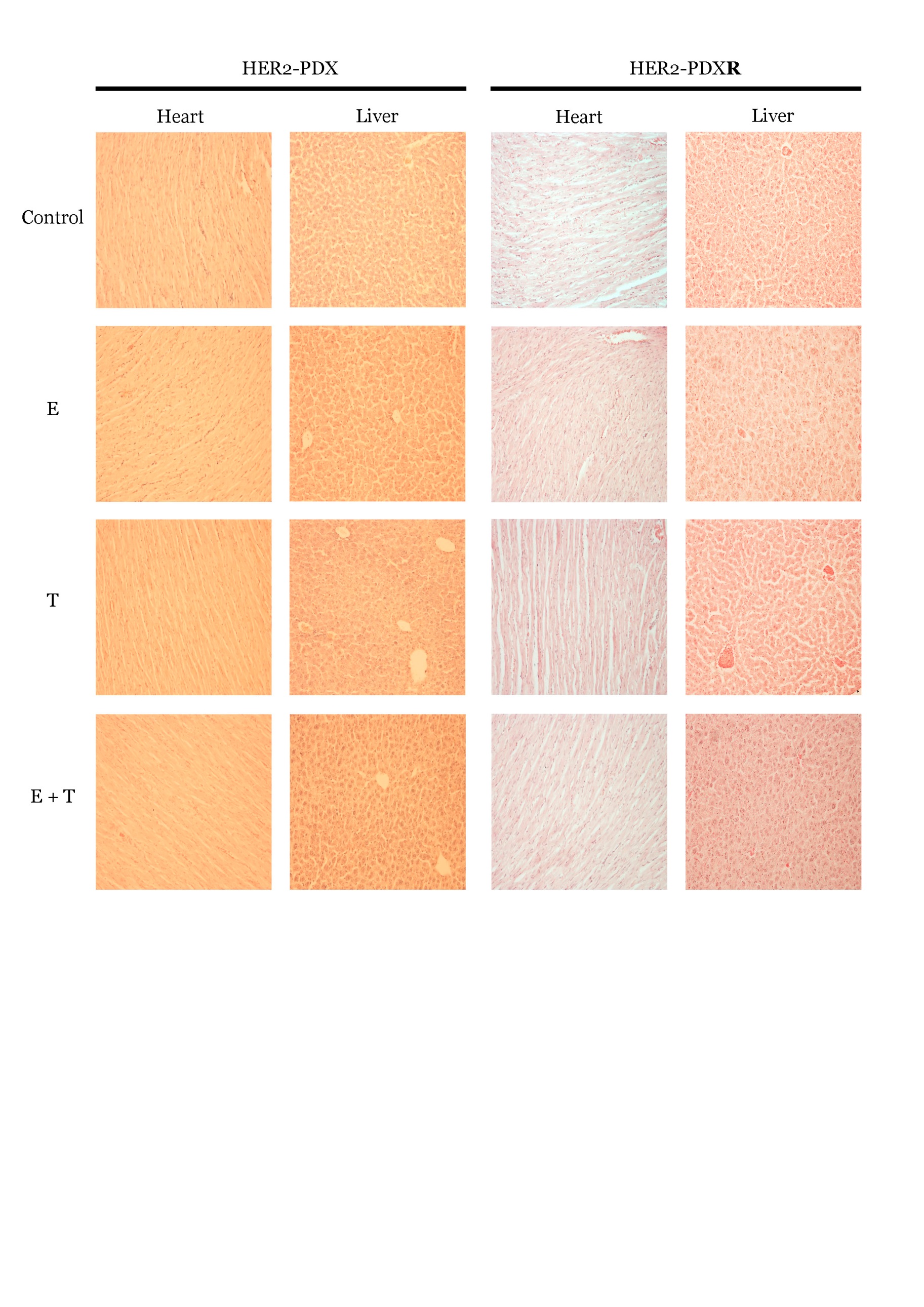
**
